# Circuit diversification in a biofilm regulatory network

**DOI:** 10.1371/journal.ppat.1007787

**Published:** 2019-05-22

**Authors:** Manning Y. Huang, Carol A. Woolford, Gemma May, C. Joel McManus, Aaron P. Mitchell

**Affiliations:** Department of Biological Sciences, Carnegie Mellon University, Pittsburgh, PA, United States of America; University of Georgia, UNITED STATES

## Abstract

Genotype-phenotype relationships can vary extensively among members of a species. One cause of this variation is circuit diversification, the alteration of gene regulatory relationships among members of a species. Circuit diversification is thought to be a starting point for the circuit divergence or rewiring that occurs during speciation. How widespread is circuit diversification? Here we address this question with the fungal pathogen *Candida albicans*, which forms biofilms rich in distinctive hyphal cells as a prelude to infection. Our understanding of the biofilm/hyphal regulatory network comes primarily from studies of one clinical isolate, strain SC5314, and its marked derivatives. We used CRISPR-based methods to create mutations of four key biofilm transcription factor genes–*BCR1*, *UME6*, *BRG1*, and *EFG1* –in SC5314 and four additional clinical isolates. Phenotypic analysis revealed that mutations in *BCR1* or *UME6* have variable impact across strains, while mutations in *BRG1* or *EFG1* had uniformly severe impact. Gene expression, sampled with Nanostring probes and examined comprehensively for *EFG1* via RNA-Seq, indicates that regulatory relationships are highly variable among isolates. Our results suggest that genotype-phenotype relationships vary in this strain panel in part because of differences in control of *BRG1* by *BCR1*, a hypothesis that is supported through engineered constitutive expression of *BRG1*. Overall, the data show that circuit diversification is the rule, not the exception, in this biofilm/hyphal regulatory network.

## Author summary

Much of what we know about microbial pathogens is derived from in-depth analysis of one or a few standard laboratory strains. This statement is especially true for the fungal pathogen *Candida albicans*, because most studies have centered on strain SC5314 and its genetically marked derivatives. Here we examine the functional impact of mutations of four key biofilm regulators across five different clinical isolates. We observe that functional impact of the mutations, based on biological phenotypes and gene expression effects, varies extensively among the isolates. Our results support the idea that gene function should be validated with multiple strain isolates. In addition, our results indicate that a core regulatory network, which comprises regulatory relationships common to multiple isolates, may be enriched for functionally relevant genes.

## Introduction

Each species has broad properties that define its members, yet individuals present diversity that reflects the events of their lineage. Although some phenotypic differences between individuals arise from single allelic differences or gene acquisitions, the vast majority represent the interplay of multiple genetic and epigenetic differences [1,2,3]. Natural variation has been measured through assays of biological phenotypes such as fitness, disease susceptibility, or cellular differentiation, and through molecular phenotypes such as the expression of sets of genes. The impact of natural variation is also manifested in genetic background effects on the phenotypes of defined mutations. Comparison of large scale gene knock-out or knock-down collections in pairs of *Saccharomyces cerevisiae* [4,5] and *Caenorhabditis elegans* [6] strains has shown that genetic background effects are widespread, affecting single gene loss-of-function phenotypes for up to 20% of genes. The implication of such studies, as proposed by Gasch and colleagues, is that network relationships between genes may vary considerably among representatives of the same species [7].

The clearest example to date in fungi of species-level natural variation in network architecture comes from Chin et al., who studied adherence in two strains of *S*. *cerevisiae* [8]. A MAP Kinase pathway (fMAPK) is required for adherence and expression of the adhesin gene *FLO11* in strain Σ1278b but not in strain S288c. Crosses between the strains indicated that the regulation of fMAPK-dependence is genetically complex, though a cloning-based rescue strategy defined one modifier locus, *RPI1*, that specifies a transcription factor. Rpi1 can bypass the fMAPK pathway through its ability to bind to the 5' region of *FLO11*, an ability enabled by the *RPI1* allele of S288c and abolished by the *RPI1* allele of Σ1278b [8]. Therefore, these two representatives of the same species rely upon distinct signaling pathways—either an fMAPK-dependent pathway or an Rpi1-dependent pathway—to control expression of *FLO11* and, ultimately, adherence [8]. Chin et al. hypothesized that the natural variation in regulatory relationships that they observed within a species, which they call "circuit diversification," is a precursor to the evolutionary rewiring and circuit divergence that is observed between species.

How prevalent is circuit diversification among members of a species? What is the extent of its impact? Here we use the fungal pathogen *Candida albicans* to address these questions. We focus on two well characterized virulence traits: its ability to grow as hyphae and to produce a biofilm [9,10]. Hyphae are tubular arrays of cells that can be hundreds of microns in length, and hypha-associated genes specify adhesins, hydrolases, and the toxin Candidalysin that together cause tissue damage [10,11]. Biofilms are multicellular surface-bound communities that produce an extracellular matrix and are recalcitrant to antimicrobial treatment [12]. Biofilms of *C*. *albicans* are rich in hyphae, and genetic studies indicate that biofilm production depends upon hyphae in vitro and in animal infection models [12]. Biofilm formation is connected to virulence because biofilm on implanted medical devices is a major source of infection [12].

Our understanding of *C*. *albicans* biofilm formation comes primarily from studies of one clinical isolate, strain SC5314, and its derivatives CAI-4, BWP17, and SN152, whose markers facilitate genetic manipulation. Among the most well characterized biofilm regulators are the transcription factors (TFs) Efg1, Bcr1, Ume6, and Brg1 ([13,14,15,16,17,18]; reviewed in [19]). A deletion mutation affecting any one can cause a biofilm defect, depending upon the precise growth conditions. All four TFs are also required under many conditions for normal hyphal formation, expression of hypha-associated genes, and virulence in animal models. These TFs are interconnected through their control of overlapping sets of target genes and of one another's expression [19].

Because biofilm production and hyphal formation have been extensively characterized, this network provides a valuable starting point for an appraisal of natural variation. Uniform network architecture may prevail among *C*. *albicans* isolates, or circuit diversification may prevail. We test these possibilities through analysis of four different single gene deletion mutations in five different *C*. *albicans* clinical isolates. Our results show that the gene expression impact of regulatory network defects is highly variable among strains, and thus argue that circuit diversification is widespread.

## Results

### Natural variation in biofilm production

Our studies employed five *C*. *albicans* clinical isolates: SC5314 (clade 1), P76067 (clade 2), P57055 (clade 3), P87 (clade 4), and P75010 (clade 11) [20,21]. SC5314 is a dermatological isolate and is the standard laboratory strain for most molecular and genetic studies; P76067, P57055, and P75010 are bloodstream isolates; P87 is an oral isolate. These strains were chosen to represent the major clades of clinical isolates and thus to capture the range of genetic diversity.

**Fig 1:**
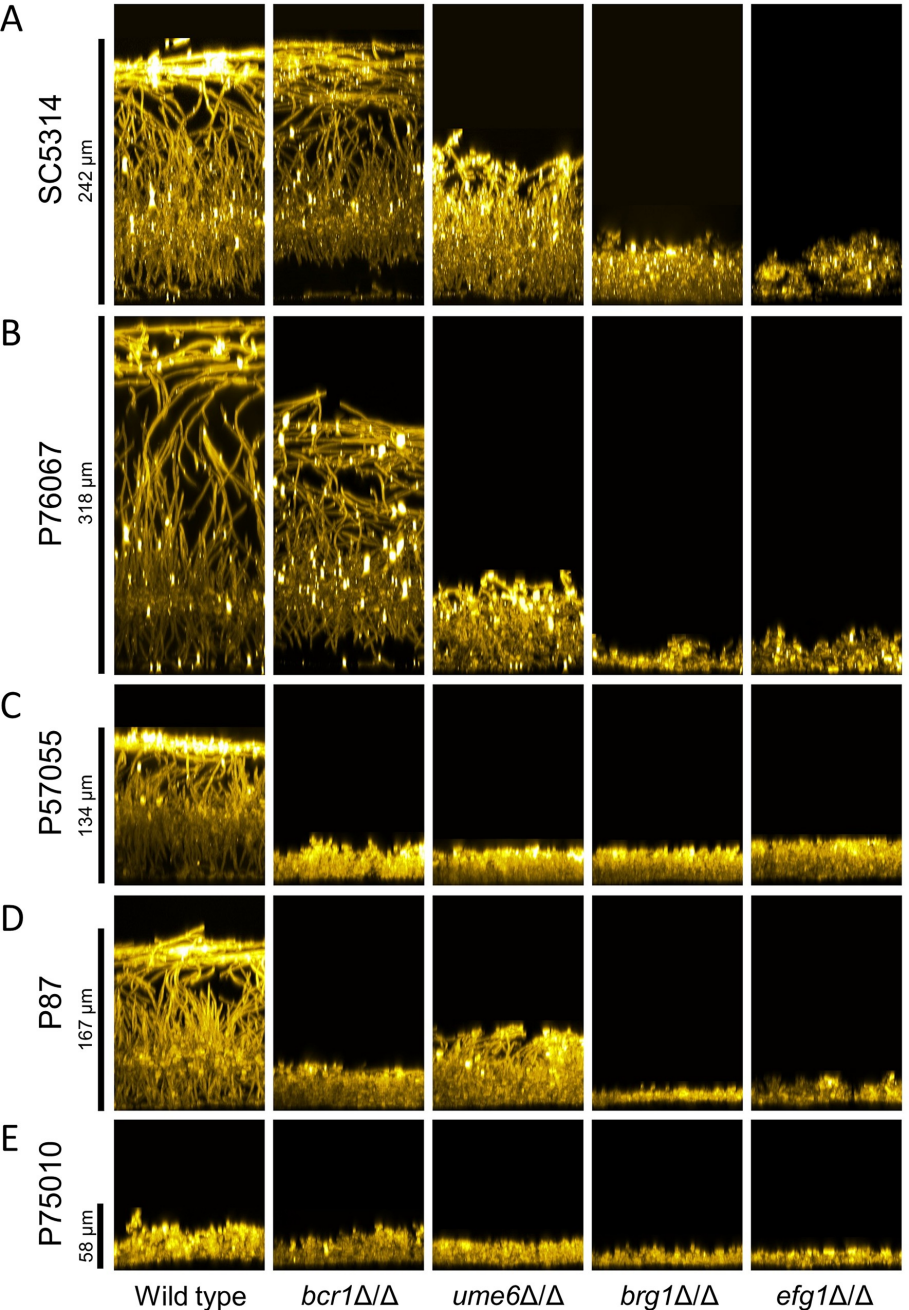
Biofilm side-view projections. Wild-type and mutant strains in each clinical isolate background were assayed for biofilm formation under *in vitro* conditions. All strains were grown on silicone squares in RPMI + 10% serum at 37°C for 24 hours. Fixed biofilms were stained using Concanavalin A, Alexa Fluor 594 conjugate, then imaged by confocal microscopy. Representative sections from each biofilm are shown; relevant genotypes are given beneath each column. Scale bars indicate the depth of the corresponding wild-type biofilm. Strain backgrounds: **A.** SC5314. **B.** P76067. **C.** P57055. **D.** P87. **E.** P75010.

Biofilm production was assayed at the end of a 24 hr incubation in RPMI+serum medium at 37 degrees. These conditions induce biofilm formation strongly in strain SC5314 (Fig 1, left column, and S1 Fig). Biofilm depth, visualized by confocal microscopy, was substantial for strains SC5314 and P76067, intermediate for strains P57055 and P87, and minimal for strain P75010 (Fig 1, left column, and S1 Fig). These results indicate that biofilm production varies among this set of isolates.

**Fig 2:**
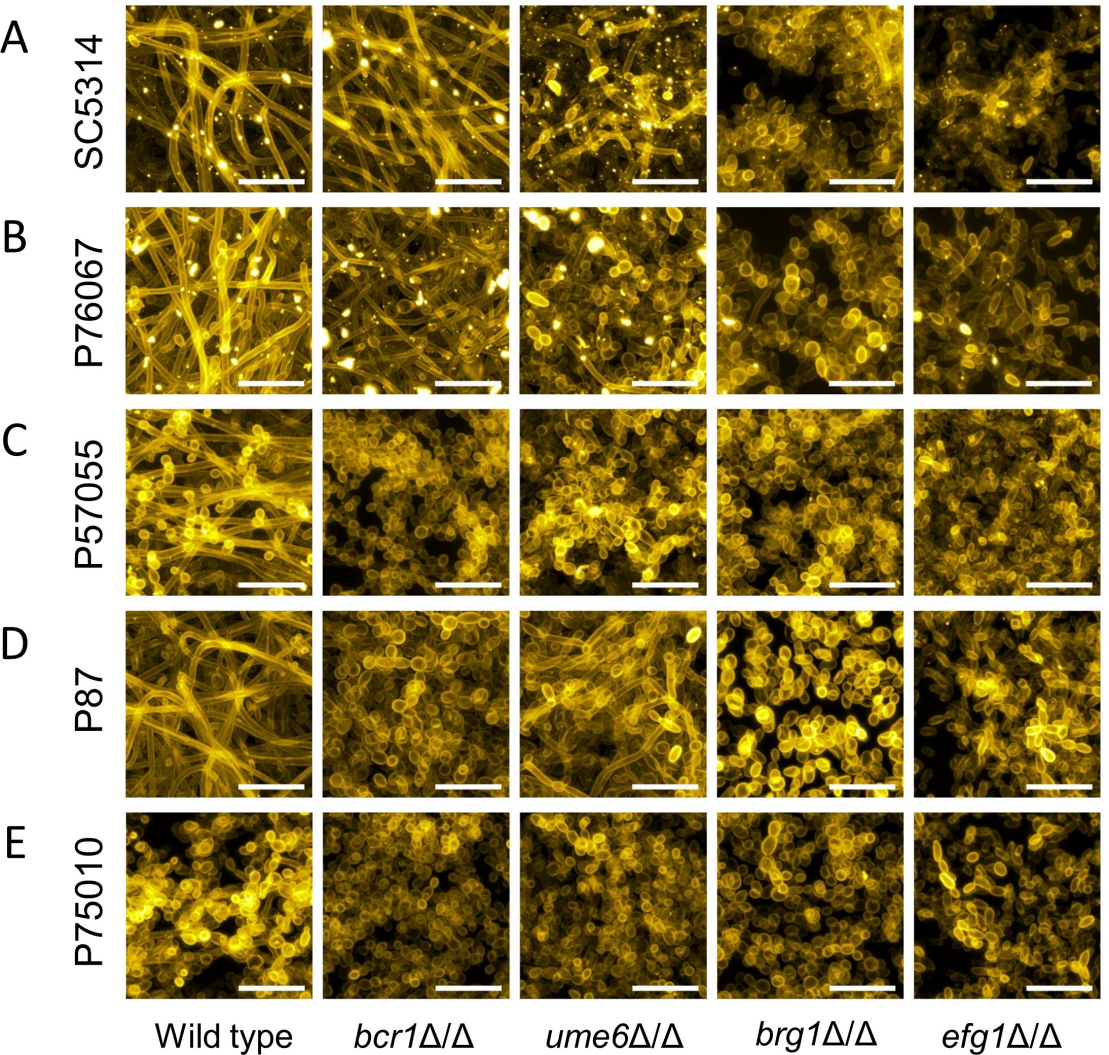
Biofilm apical-view projections. Apical views of representative sections from each clinical isolate and mutant biofilm are shown. Relevant genotypes are given beneath each column. White scale bars in each panel are 20μm in length. Projections were generated with the same datasets used in Fig 1. Strain backgrounds: **A.** SC5314. **B.** P76067. **C.** P57055. **D.** P87. **E.** P75010.

Confocal imaging was used to assay for presence of hyphae in biofilms. Side-view (Fig 1, left column) and apical (Fig 2, left column) confocal projections revealed presence of abundant hyphae in the four strong and intermediate biofilms. No hyphae were evident in the minimal biofilm produced by strain P75010. These results are consistent with the conclusion from extensive mutant analysis in the strain SC5314 background that hyphal formation is required for biofilm formation [19].

**Fig 3:**
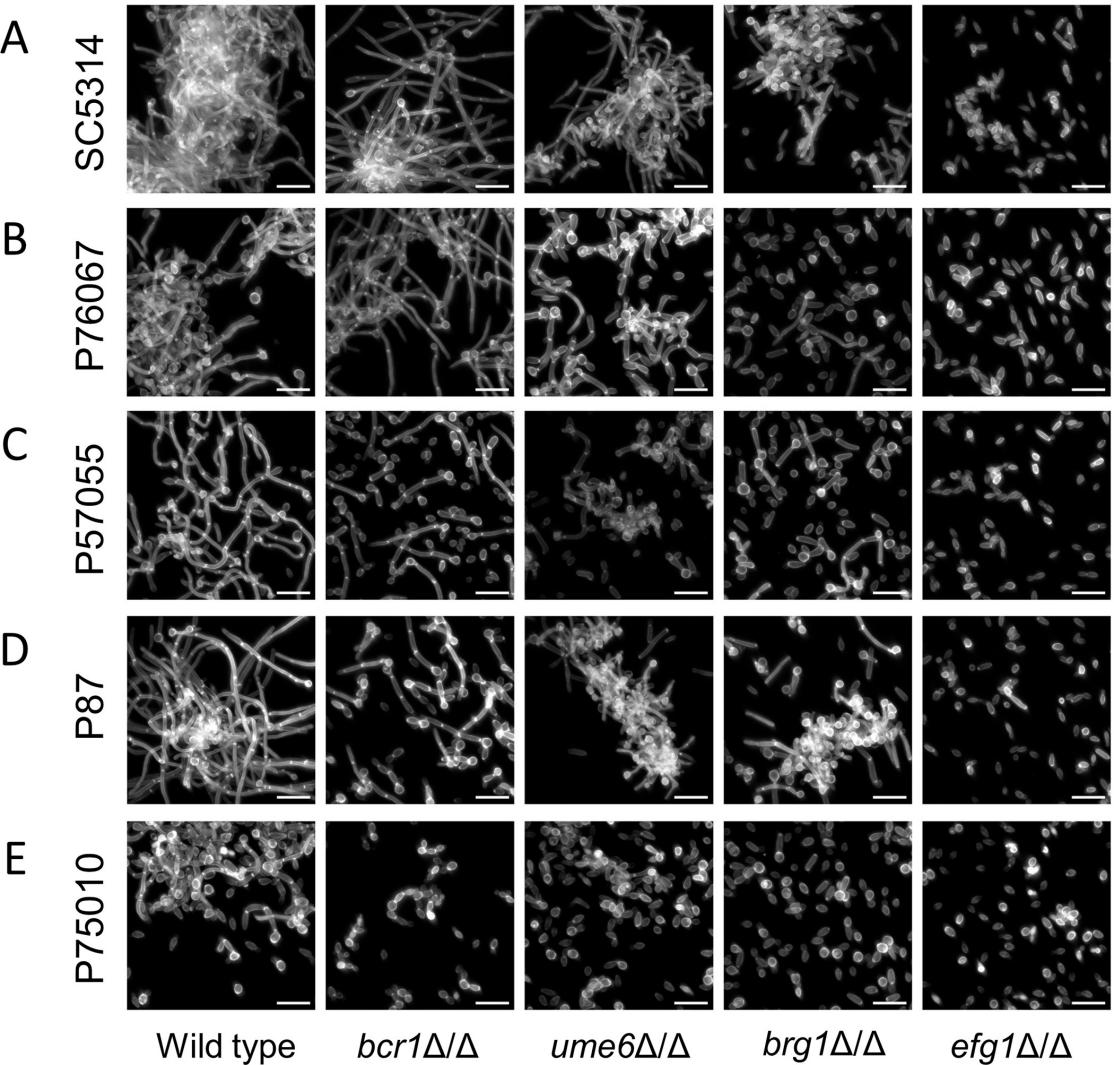
Filamentation assays. Wild-type and mutant strains of each background were assayed for filamentation under planktonic growth conditions. Strains were grown in RPMI + 10% serum at 37°C for 4 hours with shaking. Fixed cells were stained with Calcofluor-white for confocal microscopy. White scale bars in each panel are 20μm in length. Strain backgrounds: **A.** SC5314. **B.** P76067. **C.** P57055. **D.** P87. **E.** P75010.

We also assayed hyphal formation by each strain under planktonic growth conditions (4 hr, RPMI+serum medium, 37 degrees). The strong and intermediate biofilm formers produced abundant long hyphae (Fig 3, left column). The intermediate biofilm forming strain P57055 produced slightly unusual hyphae; many had bends at ~20 micron intervals. The minimal biofilm forming strain P75010 yielded infrequent hyphae under these conditions. Quantitative measurements confirmed these qualitative impressions: hyphae were less abundant, and hyphal unit cell lengths were smaller, in strain P75010 than in the strong and intermediate biofilm formers (S2 Fig). These assay results indicate that production of planktonic hyphae correlates with production of biofilm hyphae in this panel of strains.

### Genotype-phenotype relationships

To assess natural variation in genetic control over biofilm production, we created deletion mutations for each of the biofilm regulatory genes *BCR1*, *UME6*, *BRG1*, and *EFG1* in all five strains. Mutants were assayed for biofilm production under RPMI+serum growth conditions. A *bcr1*Δ/Δ mutation had little impact under these conditions on biofilm production by the two strong biofilm formers, strains SC5314 and P76067: mutant biofilm depth (Fig 1) and hyphal content (Fig 2) were comparable to those of the respective wild-type strains. However, we noted regional separation of the basal and upper biofilm layers in these mutants (Fig 1). In contrast, a *bcr1*Δ/Δ mutation impaired biofilm production by the two intermediate biofilm formers, strains P57055 and P87: biofilm depth and hyphal content were severely reduced (Figs 1 and 2). A *bcr1*Δ/Δ mutation had little effect on the weak biofilm former, strain P75010 (Figs 1 and 2). Pannanusorn et al., in pioneering studies of a set of *Candida parapsilosis* clinical isolates, also observed that impact of *bcr1*Δ/Δ mutations was highly strain-dependent in that species [22]. Our results indicate that *BCR1* is dispensable for biofilm production in some *C*. *albicans* strain backgrounds and essential for biofilm production in others.

A *ume6*Δ/Δ mutation had broad effects on biofilm production: it caused a partial or severe impairment in all of the strong and intermediate biofilm former backgrounds. Biofilm depth (Fig 1) and hyphal content (Fig 2) were reduced. Biofilm disruption by the *ume6*Δ/Δ mutation was particularly severe in the intermediate biofilm former P57055, perhaps due to the absence of biofilm hyphae (Fig 2). A *ume6*Δ/Δ mutation had little measurable effect on the weak biofilm former P75010 (Figs 1 and 2). These results show that Ume6 functional impact varies with strain background, as is the case with Bcr1.

Both *brg1*Δ/Δ and *efg1*Δ/Δ mutations caused severe impairment of biofilm production in the strong and intermediate biofilm formers. Biofilm depth was reduced to ~20 microns (Fig 1), and hyphal content was nearly or entirely eliminated (Fig 2). The mutations had little effect on the already weak biofilms formed in the strain P75010 genetic background. These results show that Brg1 and Efg1 have broad functional impact on phenotype that varies minimally with strain background.

We also assayed the effect of each mutation on production of planktonic hyphae. The results (Fig 3 and S3 Fig) correlated generally with production of biofilm hyphae (Fig 2). Reconstituted derivatives of all mutants, in which one or two copies of the deleted gene were re-introduced, regained hyphal formation ability comparable to the respective wild-type strains (S4 Fig). Interestingly, P75010 derivatives that carried the *BRG1* and *EFG1* alleles from SC5314 displayed increased hyphal production compared to P75010 (S4 and S5 Figs). These results support the conclusion that the magnitude of impact on phenotype of several of the transcription factors varies with strain background.

### Natural variation in network architecture

Results above indicate that several biofilm regulatory mutations vary in phenotypic severity among the clinical isolates. To explore this conclusion at the level of gene expression, we conducted Nanostring profiling of each wild-type and regulatory mutant strain. Growth conditions were identical to those for the hyphal induction assays. RNA levels were measured for 181 genes, including 60 genes that have been connected through function or expression to hyphae or biofilms. RNA levels in each mutant were compared to the respective wild type in order to calculate fold changes (S1 Table). The results revealed that gene regulatory relationships are strongly contingent upon strain background.

One indication of regulatory variation across strains comes from a count of the number of significantly up- or down-regulated genes in each mutant strain compared to their respective wild-type strains (Table 1). For example, in the SC5314 strain background, there were 23 genes whose RNA levels were altered significantly (≥2-fold, FDR = 0.1) by a *bcr1*Δ/Δ mutation. In the P57055 background, there were 58 genes whose RNA levels were altered significantly by a *bcr1*Δ/Δ mutation. Across all backgrounds, only 12 genes responded consistently to a *bcr1*Δ/Δ mutation ("Common" column, Table 1). The overall lack of concordance presented by *bcr1*Δ/Δ mutations was recapitulated by the other mutations: the number of responsive genes varied by a factor of 2 among strain backgrounds, and the number of shared responsive genes ("Common") was fewer than half of the number of responsive genes in any background. A similar outcome was observed if only the criterion of an FDR = 0.1 was applied without a fold-change requirement (S2 Table). These results indicate that there is substantial variation in regulatory relationships within the *C*. *albicans* species.

**Table 1:** Affected genes in each strain background.

Mutant	SC5314	P76067	P57055	P87	P75010	Common
*bcr1*Δ/Δ	23	33	58	46	57	12
*ume6*Δ/Δ	18	25	34	18	15	0
*brg1*Δ/Δ	69	35	34	27	34	11
*efg1*Δ/Δ	114	56	70	79	56	28

Gene counts are based on Nanostring data (S1 Table) with a fold-change ≥2 and FDR = 0.1. Common genes are those shared among all five strain backgrounds for each TF mutant.

**Fig 4:**
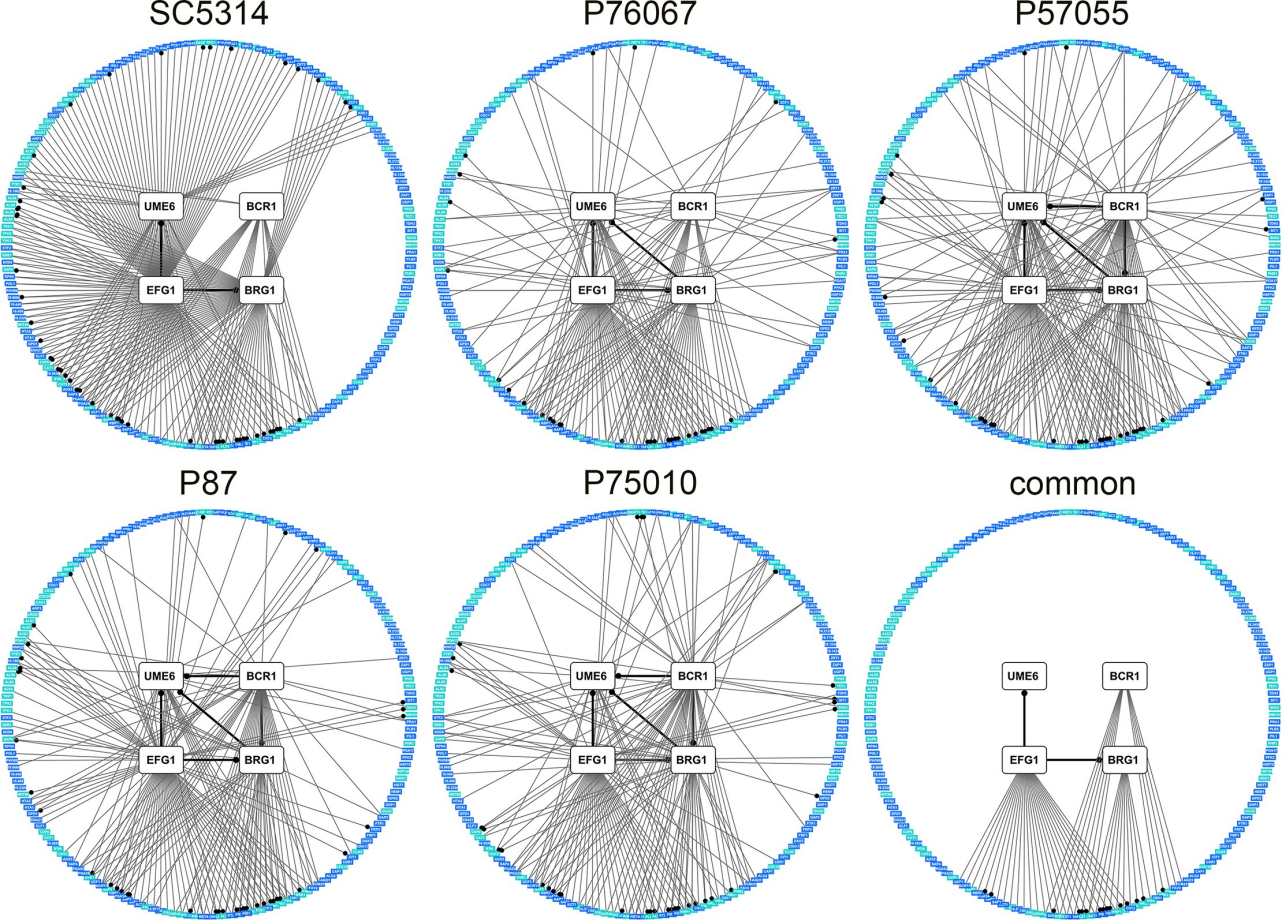
Variation in the *C*. *albicans* biofilm/hyphal regulatory network. Network diagrams are presented for each clinical isolate as well as for features shared among them ("Common"). Nodes represent genes analyzed by Nanostring, with white denoting the four TF genes, and blue and teal denoting prospective target genes. Node positions are identical across network graphs. The teal color indicates that the gene is annotated for function in biofilm or hyphal formation. A significant gene expression alteration by a TF gene mutation is denoted by an edge between two nodes; a dot on an edge indicates the connected TF was reported to bind in the upstream region of the target gene [16,23]. A significant gene expression alteration was defined as a two-fold difference in mRNA level difference between mutant and wild type, and a significant difference in mean mRNA Nanostring counts between mutant and wild type (Benjamini-Hochberg step-up procedure, FDR = 0.1). Three biological replicates were analyzed using Nanostring for all strains.

**Fig 5:**
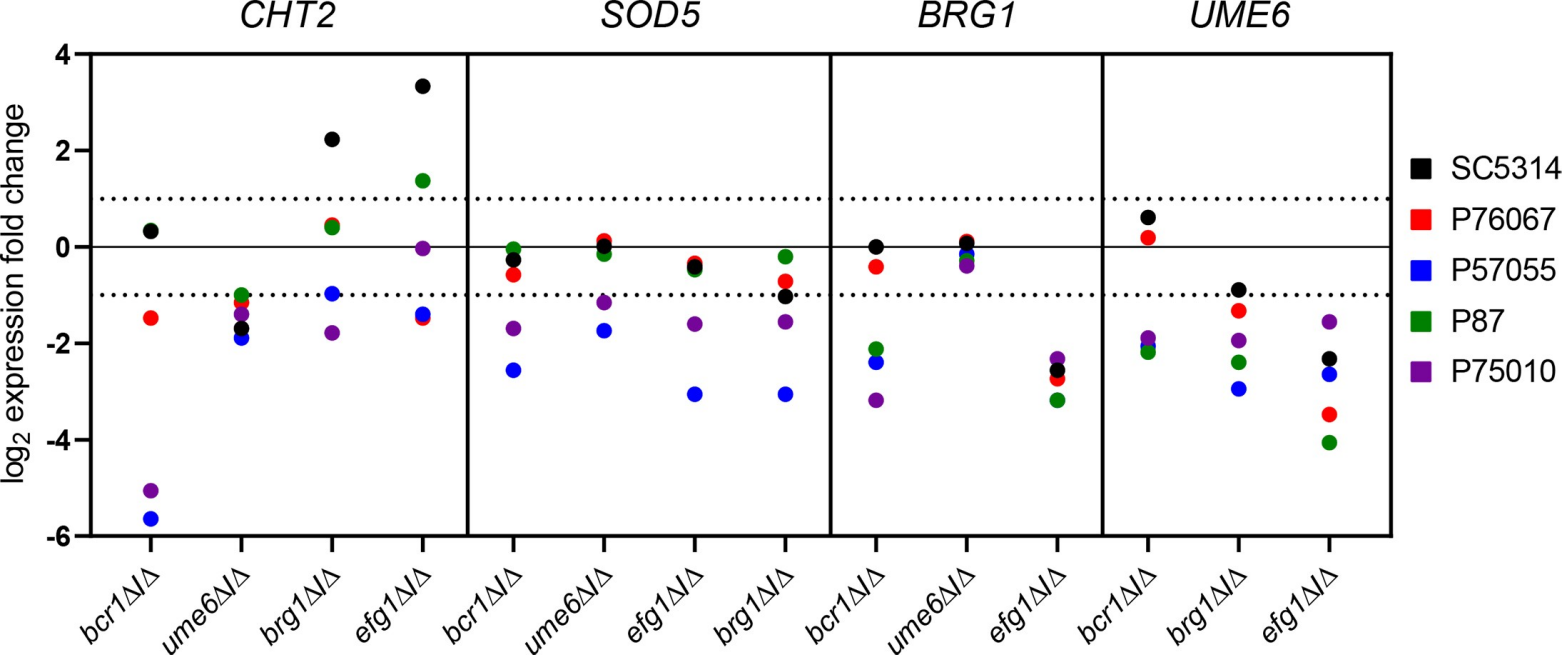
Range of TF mutant gene expression impact. Fold-change values are plotted for RNAs from the *CHT2*, *SOD5*, *BRG1*, and *UME6* genes in each TF mutant in all five strain backgrounds. Three biological replicates were analyzed using Nanostring for all strains. Data are extracted from S1 Table.

Extensive variation is also seen in the architecture of the biofilm/hyphal regulatory network defined by the mutants and their gene expression impact (Fig 4). Some genes that are annotated to hyphal formation, such as *CHT2* and *SOD5*, varied considerably with respect to strain background in their dependence on specific TFs (Fig 5). The variable response of *CHT2* was particularly noteworthy because its 5' region is bound by Bcr1 and Brg1 as shown by overlapping binding peaks centered approximately 2250 bp upstream of the start codon [16], an indication that it is a direct target of those two TFs. Interestingly, among all five isolates, no SNPs were identified in Bcr1 and Brg1 motifs in this region. An additional illustration of network variation comes from the regulation of the TF genes *BRG1* and *UME6* (Figs 4 and 5). In the two strong biofilm formers, SC5314 and P76067, Bcr1 is not required for expression of *BRG1* and *UME6*. In the intermediate and weak biofilm formers, Bcr1 is required for expression of both *BRG1* and *UME6*. These dependency relationships provide a possible explanation for the greater impact of the *bcr1*Δ/Δ mutation on gene expression and biological phenotypes in the intermediate biofilm formers than in the strong biofilm formers (Table 1; Figs 1–3). Overall, these results indicate that regulatory network architecture is strongly contingent upon strain background. In addition, the observation that many genes are dependent upon a TF in one strain background but not another is evidence for circuit diversification among these strains.

We distilled gene expression changes into a common network of regulatory relationships that are shared among all five strains (Fig 4; S1G Table). In the 181 assayed genes, 60 were annotated to GO terms related to hyphae or biofilm. Compared to regulatory relationships determined solely in SC5314, a larger proportion of the relationships defined by this common network were with these hyphae or biofilm annotated genes (p = 0.014, Fisher's exact test). The common network also trended toward enrichment for direct targets of the TFs (p = 0.083 compared to SC5314, Fisher's exact test) as defined by ChIP-Seq experiments [16,23]. These observations suggest that the common target genes found in diverse strains may give clearer functional insight into their regulators than the target genes found in any one strain.

### Genome-wide Efg1 regulon analysis

**Fig 6:**
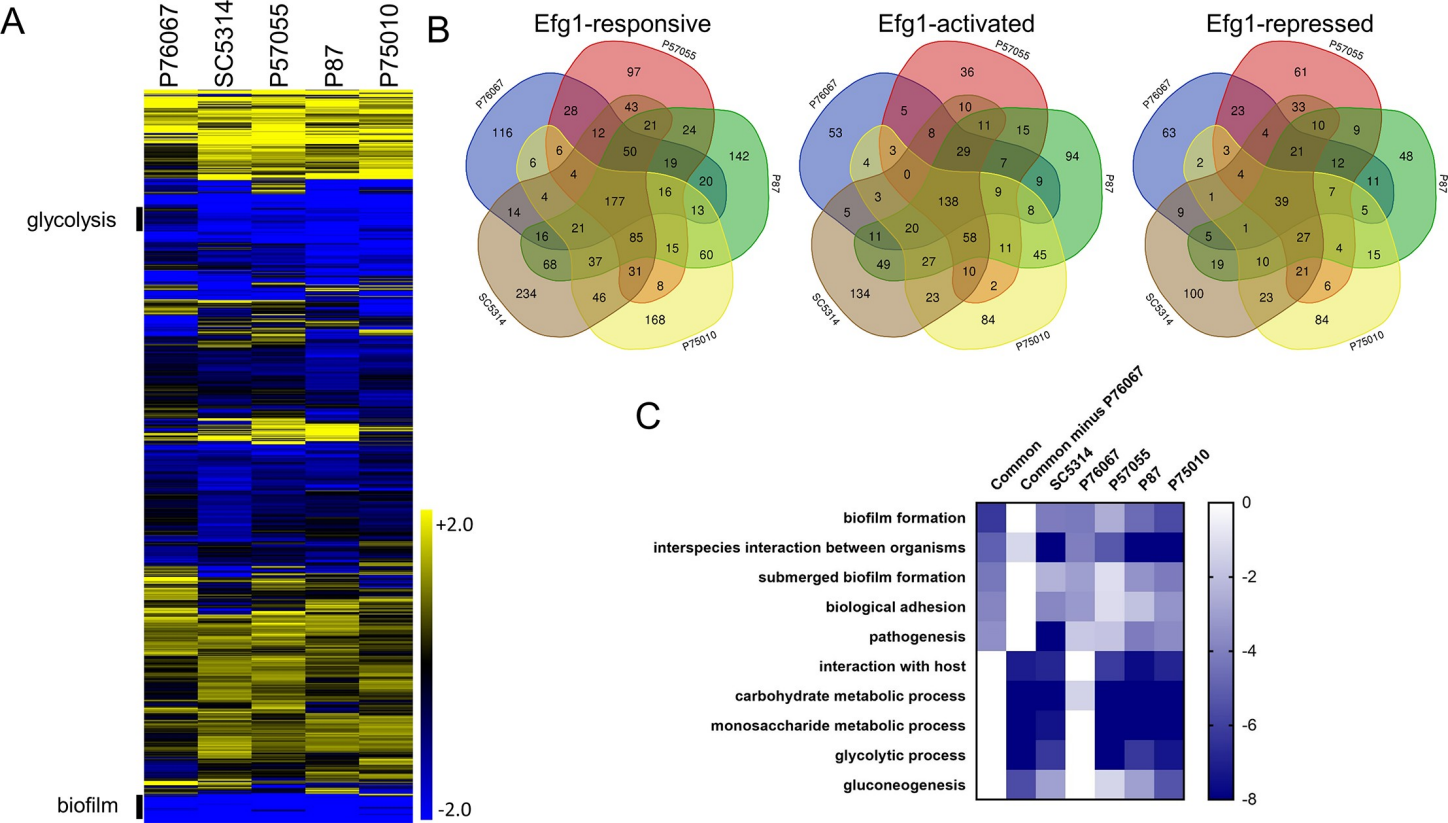
Genome-wide Efg1 regulons. Global expression was assayed using RNA-Seq. Three biological replicates were analyzed for each *efg1*Δ/Δ mutant and clinical isolate. Fold change values were determined using DeSeq2. **A.** Heatmap depicting log2 fold change in gene expression. Upper (Yellow) and lower bounds (Blue) correspond to a log2 fold change value of 2 and -2 respectively. Sections labeled glycolysis and biofilm are enriched for genes annotated for roles in glycolysis and biofilm formation respectively. **B.** Venn diagrams depicting intersection of genes dependent upon *EFG1* in each clinical isolate background. We considered all genes that were significantly differentially expressed (p<0.05, Benjamini-Hochberg adjustment), and had at least a 2 fold difference in expression between *efg1*Δ/Δ mutant and wild type. **C.** Heatmap depicting p-values from GO term analysis of sets of genes that had significantly lower expression in the *efg1*Δ/Δ mutant vs matched wild type. The analyzed sets were the set of genes dependent upon SC5134, P76067, P57055, P87, and P75010, the set of genes common to all 5 clinical isolates (SC5134 ∩ P76067 ∩ P57055 ∩ P87 ∩ P75010), and the set of genes common to all clinical isolates except P76067 (SC5134 ∩ P57055 ∩ P87 ∩ P75010—P76067). Upper (white) and lower bounds (dark blue) corresponding to a log10 P-value of 0 and -8 respectively.

For a genome-wide view of regulatory relationships among strains, we carried out RNA-Seq analysis of the five clinical isolates and their *efg1*Δ/Δ derivatives (S3 Table). Each clinical isolate was compared with its corresponding *efg1*Δ/Δ mutant in order to define Efg1-responsive genes. The gene expression impact of the *efg1*Δ/Δ mutation varied considerably among clinical isolates (Table 2). The number of Efg1-responsive genes ranged from 523 (P76067) to 864 (SC5314). Approximately 15–27% of the genes that responded to Efg1 in any one strain did not respond in any of the other four strains (Fig 6B). Many additional genes were Efg1-responsive only in a subset of genetic backgrounds (Fig 6B). Overall, these genome-wide data support the concept that gene expression targets vary considerably among *C*. *albicans* species representatives, and indicate that circuit diversification frequently affects Efg1 target genes.

**Table 2:** Efg1-regulated genes in each strain background.

Number of genes	SC5314	P76067	P57055	P87	P75010	Common
Efg1-repressed	327	210	284	243	252	39
Efg1-activated	536	312	352	541	445	138
Total Efg1-responsive	863	522	636	784	697	177
Found by ChIP-ChIP or ChIP-Seq	137	114	117	133	124	42

Gene counts are based on RNA-Seq data (S3 Table) with a fold-change ≥2 and padj < 0.5. Common genes are those shared among all five strain backgrounds.

Gene expression profiles converged on 177 core Efg1-responsive genes (21–34% of total) that were up- or down-regulated in *efg1*Δ/Δ mutants of every strain background (Fig 6B). These core Efg1-responsive genes included 138 Efg1-activated genes (i.e., down-regulated in *efg1*Δ/Δ mutants) and 39 Efg1-repressed genes (i.e., up-regulated in *efg1*Δ/Δ mutants). Core Efg1-activated genes were enriched for the GO term biofilm formation (p = 4.86e-07) (Fig 6A and 6C; S4 Table). This enrichment was greater than observed with the Efg1-activated genes of any individual strain (Fig 6A and 6C; S4 Table). Core Efg1-repressed genes were enriched for GO terms that include cell surface (p = 6.94e-07) and cell wall (p = 6.78e-07) (S4 Table). This enrichment was comparable to that observed with individual strains. We found that 24% of core Efg1-responsive genes were direct Efg1 targets, based on chromatin immunoprecipitation data [16,23], whereas 16–22% of Efg1-responsive genes in individual strains were direct targets (Table 2). Compared to the proportion of direct targets among all SC5314 Efg1-responsive genes, the proportion of direct targets among core Efg1-responsive genes was greater (p = 0.036, Fisher's exact test), though it only trended toward greater in comparisons to some other strains. Overall, these observations indicate that core Efg1-responsive genes align well with what is known about Efg1 function.

The Efg1-activated genes of several strains were enriched for carbohydrate metabolic functions (Fig 6A and 6C, S6 Fig), which are mainly glycolytic genes, as expected from prior studies [24]. However, there was no enrichment for these functions in the core Efg1-activated gene set. Their exclusion from core genes is based on properties of one strain, P76067. Examination of individual gene expression responses shows that these genes display less dependence on Efg1 for expression in strain P76067 compared to the other strains (S3 Table). Therefore, the impact of Efg1 on carbohydrate metabolic genes behaves as a quantitative trait among *C*. *albicans* isolates.

The unique Efg1-responsive genes in each strain (S3 Table) ranged from 97 (strain P57055) to 234 (strain SC5314). They were roughly split between Efg1-activated and -repressed genes (Fig 6B). We found only minor enrichments for GO terms among most of these gene sets, and no significant enrichment at all among the SC5314 strain-specific Efg1-responsive genes. Although these genes do not share distinguishing GO assignments, there are prospective functionally relevant genes among them. For example, the SC5314 *efg1*Δ/Δ mutation leads to significantly reduced expression of *SUN41*, which is required for biofilm formation [25,26]. Therefore, strain-specific Efg1-responsive genes may contribute to the mutant phenotype, but they do not reveal broad pathways that respond to Efg1 in a strain-specific manner.

### Functional impact of circuit diversification

The *BCR1-BRG1* relationship provides a simple illustration of circuit diversification: *BCR1* is required for *BRG1* expression in intermediate but not strong biofilm formers (Figs 4 and 5). We hypothesized that this regulatory difference was the reason that *BCR1* is required for biofilm production by intermediate but not strong biofilm formers under our assay conditions. Specifically, reduced *BRG1* expression may contribute to the biofilm defect of *bcr1*Δ/Δ mutants in intermediate biofilm formers, while constitutive *BRG1* expression may permit biofilm production by *bcr1*Δ/Δ mutants in strong biofilm formers.

**Fig 7:**
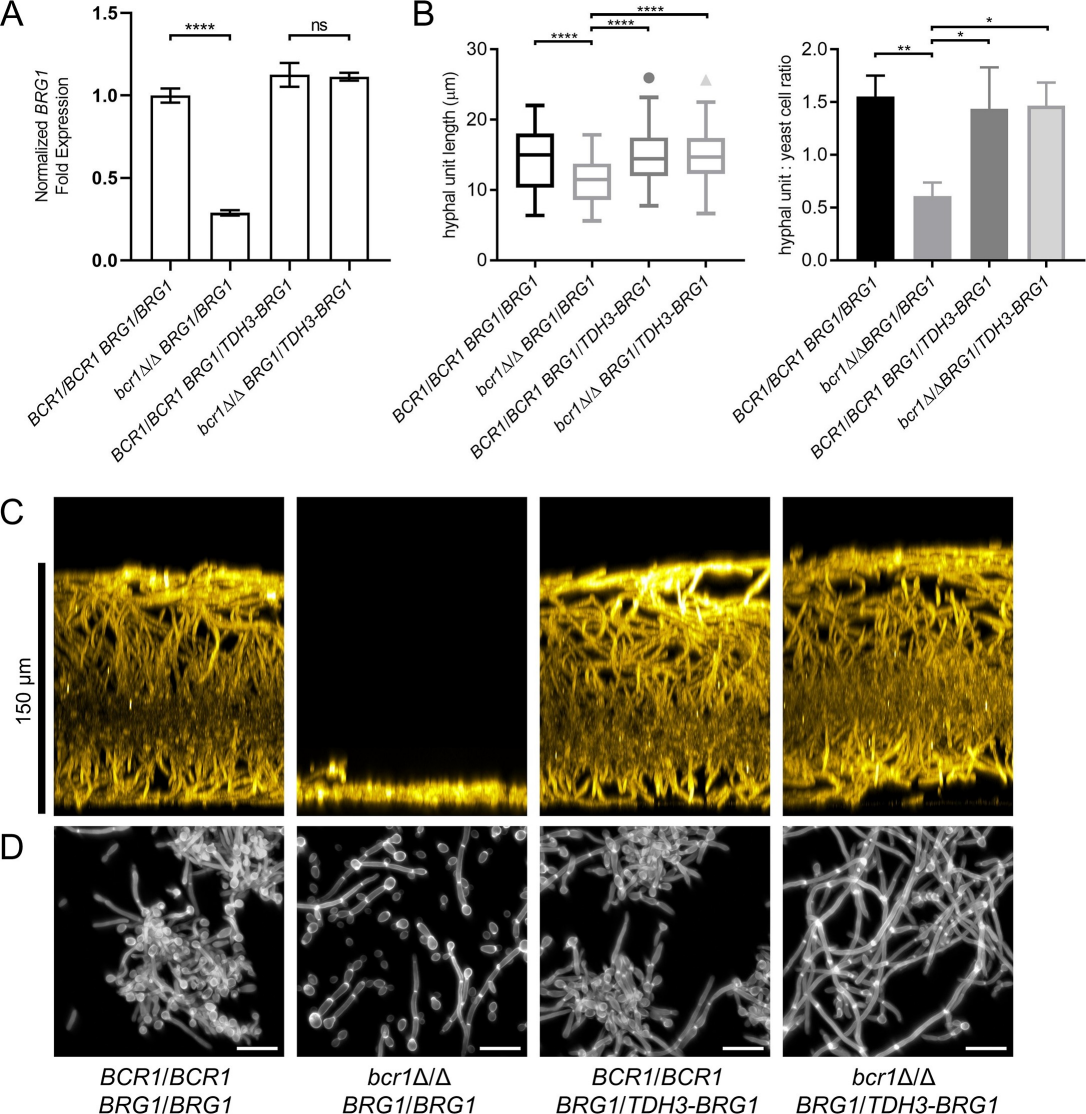
Impact of constitutive *BRG1* expression in a Bcr1-dependent strain background. Parental *BRG1/BRG1* strains and derived *BRG1/TDH3-BRG1* were assayed for planktonic hyphal formation and biofilm production in RPMI + 10% serum at 37°C. Planktonic cultures were grown for 4 hours, and biofilm cultures were grown for 24 hours. **A.** Fold change in expression of *BRG1* mRNA analyzed by Nanostring. Values shown are mean (SD). Significance is indicated above horizontal bars (Tukey-Kramer test; “****”, P < 0.0001). Three biological replicates were analyzed. **B.** Hyphal length and hypha to yeast ratios were quantified in planktonic culture samples. Values shown are mean (SD). Three technical replicates were performed for each strain. Pairs of means connected by a horizontal bar are significantly different (Tukey-Kramer test; “*”, P < 0.05; “**”, P < 0.01; “****”, P<0.0001); all unconnected pairs are not significantly different. **C.** Side-view projections of biofilms stained with ConA-Alexafluor 594 conjugate. Scale bar on left indicates depth of wild-type biofilm. **D.** Images of planktonic culture samples stained with Calcofluor-white. White scale bars in each panel are 20μm in length.

This hypothesis predicts that constitutive *BRG1* expression will permit biofilm production in a *bcr1*Δ/Δ mutant in an intermediate biofilm former. We tested this hypothesis with strain P57055, an intermediate biofilm former that transforms efficiently. We fused one allele of *BRG1* with the *TDH3* promoter in P57055 *BCR1/BCR1 BRG1/BRG1* and *bcr1*Δ/Δ *BRG1/BRG1* strains to create *BRG1/TDH3-BRG1* derivatives. We then compared four strains of genotypes *BCR1/BCR1 BRG1/BRG1*, *bcr1*Δ/Δ *BRG1/BRG1, BCR1/BCR1 BRG1/TDH3-BRG1*, and *bcr1*Δ/Δ *BRG1/TDH3-BRG1*. Using Nanostring, we confirmed that *BRG1* RNA levels were Bcr1-dependent in the *BRG1/BRG1* strains and Bcr1-independent in the *BRG1/TDH3-BRG1* strains (Fig 7A). Although the *TDH3* promoter is often used for overexpression, in this case it did not yield greatly elevated *BRG1* expression. As predicted by the hypothesis, biofilm production was also Bcr1-dependent in the *BRG1/BRG1* strains and Bcr1-independent in the *BRG1/TDH3-BRG1* strains (Fig 7C). As a further functional test of the hypothesis, we examined planktonic hyphal formation. In the P57055 background, the *bcr1*Δ/Δ mutant had reduced length of hyphal cell compartments and a reduced ratio of hyphae to yeast cells (Fig 7B and 7D). In the P57055 *BRG1/TDH3-BRG1* derivatives, the *bcr1*Δ/Δ mutant did not display these phenotypes (Fig 7B and 7D). Therefore, the phenotypic impact of a *bcr1*Δ/Δ mutation in the P57055 background depends upon the *BCR1-BRG1* regulatory relationship.

What is the mechanism behind divergent dependence of *BRG1* expression on Bcr1? One hypothesis is that the cis regulatory elements of *BRG1* alleles may contain SNPs that allow Bcr1-independent *BRG1* expression in some strains but not others. We tested this hypothesis by constructing P57055 *bcr1*Δ/Δ mutant strains carrying *BRG1*^SC5314^ or *BRG1*^P57055^ alleles at the *MDR1* locus. These alleles contained 1642 bp of the *BRG1* upstream region and 712 bp of the *BRG1* downstream region. Ectopic expression of *BRG1*^SC5314^ and *BRG1*^P57055^ in this manner complemented the hyphal formation defect of a P57055 *brg1*Δ/Δ mutant strain, demonstrating that the cis contexts captured in these regions were sufficient for *BRG1* expression and function (S7A Fig). However, ectopic expression of *BRG1*^SC5314^ or *BRG1*^P57055^ in the P57055 *bcr1*Δ/Δ mutant failed to rescue hyphal formation (S7B Fig and S5 Table). Furthermore, strains expressing *BRG1*^SC5314^ were not significantly different from strains expressing *BRG1*^P57055^ in hyphal formation capacity. We conclude then that the cause of Bcr1-independent *BRG1* expression in SC5314 does not lie solely in cis regulatory element SNPs carried in these allelic segments.

## Discussion

Our studies address whether genetic regulatory relationships are uniform within the species *C*. *albicans*. We approached the problem through measurement of biological phenotypes and gene expression changes that result from mutations in each of four TF genes in the biofilm/hyphal regulatory network. Two of the TF gene mutations, *bcr1*Δ/Δ and *ume6*Δ/Δ, had variable phenotypic impact among the strains. These mutations also had variable gene expression impact; an outcome that might have been predicted from phenotypic variation. The other two TF mutations, *brg1*Δ/Δ and *efg1*Δ/Δ, had uniform phenotypic impact, yet still had highly variable gene expression impact. These observations argue that circuit diversification–variation in regulator-target relationships within a species–is prevalent for this biofilm/hyphal regulatory network.

The traits we examined, biofilm production and hyphal formation, are known to vary quantitatively among *C*. *albicans* isolates [20,21,27,28,29]. Hence it seemed reasonable that gene expression impact of key biofilm/hyphal regulators would vary as well. We were nonetheless struck by the extent of strain-specific gene expression changes we observed; only about half of the gene expression response to a mutation in any one strain was shared among the other four strains. The fact that even the mutation with the strongest and most uniform biological phenotypes, *efg1*Δ/Δ, caused variable gene expression impact across strains is especially noteworthy, because large scale dual-strain comparisons of loss-of-function defects have relied on biological phenotypes [4,5,6]. Our results argue that biological phenotype measurements may underestimate the difference in impact of a mutation in two different strains.

What sorts of variation do we see in TF-target gene relationships? Regulation of *SOD5*, *BRG1*, and *UME6* (Fig 5) represents one frequent pattern: expression of each is down-regulated in a mutant, such as *bcr1*Δ/Δ, in some strains but not others. Analogous observations were made with expression of *S*. *cerevisiae FLO11* and its control by the fMAPK pathway by Chin and colleagues [8]. The regulation of *CHT2* is more complex, for example in its response to Efg1. It is up-regulated in an *efg1*Δ/Δ mutant in SC5314, as shown previously [30], but it is down-regulated in *efg1*Δ/Δ mutants of other strains. Efg1 is known to function as an activator at some promoters and a repressor at others [31,32] in strains derived from SC5314. However, our results raise the possibility that Efg1 may function as a repressor or an activator at a single promoter, depending upon the strain background. These examples illustrate strain-dependent differences in TF-target gene relationships that are indicative of circuit diversification.

Variation in biofilm/hyphal network architecture has clear functional impact, as illustrated by strain differences in the *BCR1-BRG1* relationship. A *bcr1*Δ/Δ mutation had little effect on biofilm production or *BRG1* expression in two strong biofilm formers, and caused a severe defect in both biofilm production and *BRG1* expression in two intermediate biofilm formers. Because *BRG1* was required for biofilm production in all strains, we considered that differences in *BCR1*-dependence of biofilm production may arise from differences in *BCR1*-dependence of *BRG1* expression. This hypothesis was supported by the finding that constitutive *BRG1* expression eliminated *BCR1*-dependence of biofilm production in an intermediate biofilm former. Prior studies have shown that Bcr1 and Brg1 have considerable functional overlap: among 252 direct Bcr1 target genes identified by ChIP-seq, 194 are Brg1 direct targets as well [16]. Overlap of target genes may be the reason that *BCR1* is required for biofilm formation only when *BRG1* levels are low. We cannot find strain differences in the Bcr1 binding sites upstream of *BRG1*. Furthermore, cis-regulatory elements of the *BRG1*^SC5314^ allele were not sufficient for *BRG1* function in the P57055 *bcr1*Δ/Δ mutant strain. We infer that variation in the *BCR1-BRG1* relationship arises from differences in trans-acting factors that can compensate for absence of Bcr1. This inference is consistent with the conclusion from many studies the bulk of gene expression variance between individuals arises from differences in trans-acting gene products [1].

Glycolytic genes provide an example of a functionally related group of genes that vary in strength of connection to biofilm regulator Efg1 (Fig 6, S6 Fig). In most strains, the *efg1*Δ/Δ mutation caused a severe reduction in glycolytic gene expression. In contrast, in strain P76067 the *efg1*Δ/Δ mutation caused a mild reduction in glycolytic gene expression. Inspection of the RNA-Seq data shows that expression of *GAL4*, an activator of glycolytic genes [33], is strongly reduced in most *efg1*Δ/Δ mutants but only mildly reduced in the P76067-derived *efg1*Δ/Δ mutant. Efg1 does not bind directly to the *GAL4* upstream region [16,23]. Therefore, this example of circuit diversification also seems to arise from differences in activity of trans-acting factors that, in this case, compensate for absence of Efg1.

Our data provide the first view of *C*. *albicans* natural variation from the perspective of gene expression profiles, and several manifestations of strain variation are evident. For example, compared to SC5314, all isolates had significantly increased RNA levels for various cell wall-related genes (S6C Table). Also, higher *BCR1* and *BRG1* RNA levels among isolates correlate with lower RNA levels for ribosome-related genes (S6A Table). These correlations may reflect natural variation in TOR pathway activity, which is known to promote ribosome biogenesis and inhibit Bcr1-dependent adhesin expression [34]. Although high resolution trait mapping is not yet feasible for *C*. *albicans*, a candidate gene-based approach could unravel the causes for these strain differences and their functional consequences.

A valuable practical application of multi-strain analysis is the distillation of a common set of genetic regulatory relationships. This outcome was suggested by our small-scale Nanostring profiling, but was most clearly documented through genome-wide analysis of Efg1-responsive genes. Specifically, the common Efg1-activated gene set was significantly enriched for biofilm-related genes, and trended toward enrichment for direct Efg1 target genes, compared to any individual strain's Efg1-activated genes. The common Efg1-repressed gene set was enriched for cell-surface related genes, an enrichment that was not found among Efg1-repressed gene sets for individual strains. These outcomes argue that multi-strain analysis of mutants is significant both for the validation of conclusions across multiple species representatives, and for its ability to narrow a panel of responsive genes to those with a strong connection to relevant biological processes.

## Methods

### Strains and media

The following *C*. *albicans* clinical isolate strains were obtained through BEI Resources, NIAID, NIH: *Candida albicans*, Strain P76067, NR-29442; *Candida albicans*, Strain P57055, NR-29439; *Candida albicans*, Strain P87, NR-29453; *Candida albicans*, Strain P75010, NR-29437. All strains and mutants were maintained in 15% glycerol stocks stored at -80°C. Prior to all experiments, strains were grown on YPD (2% Bacto Peptone, 2% dextrose, 1% yeast extract) for 2 days at 30°C, and then cultured overnight in liquid YPD at 30°C with shaking. Transformants were selected on YPD + 400 μg/ml nourseothricin or complete synthetic media (CSM) (2% dextrose, 1.7% Difco yeast nitrogen base with ammonium sulfate and auxotrophic supplements). For phenotypic assays, strains were grown in liquid RPMI-1640 Media (Sigma-Aldrich, Inc., St. Louis) adjusted to pH 7.4 and supplemented with 10% fetal bovine serum (Atlanta Biologicals, Inc., Flowery Branch). A full list of the strains used in this study is provided under supplemental files (S7 Table).

### Primers and plasmid construction

All primers and plasmids used in this study are provided under supplemental files (S8 Table).

We previously demonstrated that the use of repeat flanked selectable markers allowed for CRISPR-Cas9 induced marker excision in subsequent manipulations [35]. To adapt the *NAT1* marker for marker recycling with this method, we generated vectors containing *NAT1* inserted respectively at the BamHI (pMH05) and XmaI (pMH06) restriction sites in the plasmid YEp24 backbone [36].

To generate plasmid pMH05, the *NAT1* marker was amplified from plasmid pNAT [37] using primers “BamHI_YEp24_H+AdapN/F” and “BamHI_YEp24_H+AdapN/R”. An aliquot of plasmid YEp24 was then digested with BamHI, and digest products were transformed alongside the *NAT1* PCR product into the *Saccharomyces cerevisiae* strain BJ8918 with selection on synthetic media lacking uracil to allow gap repair of the digested YEp24 vector with the *NAT1* PCR product [38]. The resulting vector was recovered from Ura+ transformants using a Zymoprep Yeast Plasmid Miniprep II Kit (Zymo Research, Irvine) and correct integration of *NAT1* at the BamHI restriction site was verified by PCR.

To generate plasmid pMH06, the *NAT1* marker was amplified from plasmid pNAT using primers “XmaI_YEp24_H+AdapN/F” and “XmaI_YEp24_H+AdapN/R”. An aliquot of plasmid YEp24 was then digested with XmaI, and digest products were transformed alongside the *NAT1* PCR product into the *Saccharomyces cerevisiae* strain BJ8918 with selection on synthetic media lacking uracil to allow for gap repair of the digested YEp24 vector with the *NAT1* PCR product. The resulting vector was recovered from Ura+ transformants using a Zymoprep Yeast Plasmid Miniprep II Kit and correct integration of *NAT1* at the XmaI restriction site was verified by PCR.

### Auxotrophic strain construction

To increase the number of available markers, *HIS1* was deleted in strains P76067, P57055, P87, P75010, and SC5314 using the transient CRISPR-Cas9 system [37]. Each strain was transformed with approximately 1 μg Cas9 DNA cassette, 1 μg CaHIS1 sgRNA DNA cassette, and 3 μg *his1*Δ::*r3NAT1r3* repair template. The Cas9 DNA cassette was amplified by PCR from plasmid pV1093 as previously described [37,39]. The CaHIS1 sgRNA DNA cassette was generated using split-joint PCR using previously described protocols with the primers “CaHIS1 sgRNA/F” and “CaHIS1 SNR52/R” [37]. The *his1*Δ::*r3NAT1r3* repair template was constructed in two sections using previously described protocols [35]. The first section was amplified from plasmid pMH05 using primers “HIS1 del rNATrBamHI/F” and “NAT1 CRIME/R”. The second section was amplified from plasmid pMH06 using primers “NAT1 CRIME/F” and “HIS1 del rNATrXmaI/R”. Recombination between these two sections yields the full length *his1*Δ::*r3NAT1r3* repair template following transformation.

Transformants were selected for nourseothricin resistance, and subsequently replica plated onto CSM lacking histidine to screen for a His- phenotype. Deletion of *HIS1* in candidate transformants was verified by PCR from genomic DNA using primers “CaHIS1 Check/F” and “CaHIS1 Check Int/R” for absence of the *HIS1* ORF, and using primers “CaHIS1 Check/F” and “NAT1 Check/R” for presence of the *NAT1* marker at the *his1*Δ locus.

### Transcription factor mutant strain construction

To delete *BCR1*, the *his1*Δ strains of each background were transformed with approximately 1 μg Cas9 DNA cassette, 1 μg BCR1-2 sgRNA DNA cassette, 1 μg NAT1-2 sgRNA DNA cassette, and 3 μg *bcr1*Δ::*r1HIS1r1* repair template. Inclusion of the NAT1-2 sgRNA DNA cassette targets a Cas9 mediated double stranded break to the repeat flanked *NAT1* marker at the *his1*Δ::*r3NAT1r3* locus. The segment of vector YEp24 backbone between BamHI and XmaI constitutes the repeats flanking the NAT1 marker. We refer to these repeats as “r3”. The BCR1-2 sgRNA DNA cassette was generated using split-joint PCR with the primers “sgRNA/F BCR1-2” and “SNR52/R BCR1-2”. The NAT1-2 sgRNA DNA cassette was generated using split-joint PCR with the primers “sgRNA/F NAT1-2” and “SNR52/R NAT1-2”. The *bcr1*Δ::*r1HIS1r1* repair template was generated in two parts. The first was amplified from plasmid pMH01 using primers “HIS1 CRIME/F” and “BCR1 del KpnI-rHIS1r/R”, and the second was amplified from plasmid pMH02 using primers “BCR1 del SapI-rHIS1r/F” and “HIS1 CRIME/R”.

Recombination between the direct repeats excises the marker, rendering the strain nourseothricin sensitive and leaving only a single copy of the repeat (*r3*) at the recycled locus [35]. Transformants were selected on CSM medium lacking histidine, and replica plated onto YPD + nourseothricin plates to screen for nourseothricin sensitivity. Candidate colonies were further genotyped by PCR using primers “BCR1 check up/F” and “BCR1 check int/R” for absence of the *BCR1* ORF, and using primers “BCR1 check up/F” and “CdHIS1 Check Int/R” for presence of the *HIS1* marker at the *bcr1*Δ locus.

To delete *UME6*, the *his1*Δ strains of each background were transformed with approximately 1 μg Cas9 DNA cassette, 1 μg UME6 sgRNA DNA cassette, 1 μg NAT1-2 sgRNA DNA cassette, and 3 μg *ume6Δ*::*r1HIS1r1* repair template. The UME6 sgRNA DNA cassette was generated using split-joint PCR with the primers “sgRNA/F UME6” and “SNR52/R UME6”. The *ume6*Δ::*r1HIS1r1* repair template was generated in two parts. The first was amplified from plasmid pMH01 using primers “HIS1 CRIME/F” and “UME6 del KpnI-rHIS1r/R”, and the second was amplified from plasmid pMH02 using primers “UME6 del SapI-rHIS1r/F” and “HIS1 CRIME/R”. Transformants were selected on CSM media lacking histidine, and replica plated onto YPD + nourseothricin plates to screen for nourseothricin sensitivity. Candidate colonies were further genotyped by PCR using primers “UME6 check up/F” and “UME6 check int/R” for absence of the *UME6* ORF, and using primers “UME6 check up/F” and “CdHIS1 Check Int/R” for presence of the *HIS1* marker at the *ume6*Δ locus.

To delete *BRG1*, the *his1*Δ strains of each background were transformed with approximately 1 μg Cas9 DNA cassette, 1 μg BRG1 sgRNA DNA cassette, 1 μg NAT1-2 sgRNA DNA cassette, and 3 μg *brg1*Δ::*r1HIS1r1* repair template. The BRG1 sgRNA DNA cassette was generated using split-joint PCR with the primers “sgRNA/F BRG1” and “SNR52/R BRG1”. The *brg1*Δ::*r1HIS1r1* repair template was generated in two parts. The first was amplified from plasmid pMH01 using primers “HIS1 CRIME/F” and “BRG1 del rHISr-KpnI/R”, and the second was amplified from plasmid pMH02 using primers “BRG1 del rHISr-SapI/F” and “HIS1 CRIME/R”. Transformants were selected on CSM media lacking histidine, and replica plated onto YPD + nourseothricin plates to screen for nourseothricin sensitivity. Candidate colonies were further genotyped by PCR using primers “BRG1 check up/F” and “BRG1 check int/R” for absence of the *BRG1* ORF, and using primers “BRG1 check up/F” and “CdHIS1 Check Int/R” for presence of the *HIS1* marker at the *brg1*Δ locus.

Transformations to delete *BRG1* yielded no colonies in the P87 background using this method. To isolate *brg1*Δ mutants in this background, a repair template with extended homology was employed. This cassette was generated in two pieces, using PCR from the genomic DNA of an SC5314 *brg1*Δ::*r1HIS1r1* strain. Primers “BRG1 FarUp/F” with “HIS1 CRIME/R” were used for the first piece, and “HIS1 CRIME/F” and “BRG1 FarDown/R” were used for the second piece.

To delete *EFG1*, the *his1*Δ strains of each background were transformed with approximately 1 μg Cas9 DNA cassette, 1 μg EFG1-2 sgRNA DNA cassette, 1 μg NAT1-2 sgRNA DNA cassette, and 3 μg *efg1*Δ::*r1HIS1r1* repair template. The EFG1 sgRNA DNA cassette was generated using split-joint PCR with the primers “sgRNA/F EFG1” and “SNR52/R EFG1”. The *efg1*Δ::*r1HIS1r1* repair template was generated in two parts. The first was amplified from plasmid pMH01 using primers “HIS1 CRIME/F” and “EFG1 del rHIS1r-KpnI/R”, and the second was amplified from plasmid pMH02 using primers “EFG1 del rHIS1r-SapI/F” and “HIS1 CRIME/R”. Transformants were selected on CSM media lacking histidine, and replica plated onto YPD + nourseothricin plates to screen for nourseothricin sensitivity. Candidate colonies were further genotyped by PCR using primers “EFG1 check up/F” and “EFG1 check int/R” for absence of the *EFG1* ORF, and using primers “EFG1 check up/F” and “CdHIS1 Check Int/R” for presence of the *HIS1* marker at the *efg1*Δ locus.

To generate strains constitutively expressing *BRG1*, a *NAT1-pTDH3* cassette containing flanking homology to the *BRG1* upstream region was amplified using primers “BRG1 OE/F” and “BRG1 OE/R” from plasmid CJN542 [40]. The P57055 WT and P57055 *bcr1*Δ mutant were then transformed with 3 μg of this *NAT1-pTDH3* cassette, 1 μg of Cas9, and 1 μg of P-BRG1 sgRNA DNA cassette. The P-BRG1 sgRNA cassette was generated using split-joint PCR with primers “sgRNA/F P-BRG1” and “SNR52/R P-BRG1”. Transformants were selected on YPD + nourseothricin plates for the resistant phenotype, and were genotyped by PCR using primers “BRG1 Check Up/F” and “BRG1 Check Int/R” for the presence of one copy of the native *BRG1* promoter, and “NAT1 CRIME/F” and “BRG1 Check Int/R” for presence of the *NAT1*-*pTDH3* cassette in the *BRG1* promoter region.

### Reconstituted strain construction

To validate the construction of our TF deletion mutants, we reintroduced a copy of the SC5314 allele of each TF at the TF deletion locus using our concatemer assembly method [41].

A *BCR1* cassette was amplified from SC5314 genomic DNA using primers “BCR1 check up/F” and “BCR1 3’R->pNAT 5’/R”, containing concatenating homology to a *NAT1* marker. The SC5314 *BCR1* allelic segment amplified by these primers contains 277 bp of the *BCR1* upstream region and 399 bp of the *BCR1* downstream region. A *NAT1* marker was then amplified from pNAT using “pNAT for adap/F” and “pNAT 3’R->BCR1down/R”. As no colonies were recovered from the P75010 using these cassettes, A *NAT1* marker with extended homology was amplified from strain MH351 gDNA using “pNAT for adap/F” and “BCR1 fardown/R”.

A *UME6* cassette was amplified from plasmid pSG1-UME6 (provided by K. Lagree) containing a SC5314 *UME6* allele using primers “UME6 Check Up/F” and “UME6 3’R->pNAT 5’/R”, containing concatenating homology to a *NAT1* marker. The SC5314 *UME6* allelic segment amplified by these primers contains 403 bp of the *UME6* upstream region and 399 of the *UME6* downstream region. A *NAT1* marker was then amplified from pNAT using “pNAT for adap/F” and “pNAT 3’R->UME6down/R”.

A *BRG1* cassette was amplified from plasmid pCW1071 containing a SC5314 *BRG1* allele using primers “BRG1 Check Up/F” and “BRG1 3’R->pNAT 5’/R”, containing concatenating homology to a *NAT1* marker. The SC5314 *BRG1* allelic segment amplified by these primers contains 407 bp of the *BRG1* upstream region and 400 bp of the *BRG1* downstream region. A *NAT1* marker was then amplified from pNAT using “pNAT for adap/F” and “pNAT 3’R->BRG1down/R”.

An *EFG1* cassette was amplified from plasmid pCW861 containing a SC5314 *EFG1* allele using primers “EFG1 Check Up/F” and “EFG1 3’R->pNAT 5’/R”, containing concatenating homology to a *NAT1* marker. The SC5314 *EFG1* allelic segment amplified by these primers contains 153 bp of the *EFG1* upstream region and 401 bp of the *EFG1* downstream region. A *NAT1* marker was then amplified from pNAT using “pNAT for adap/F” and “pNAT 3’R->EFG1down/R”.

The TF-containing cassette and corresponding *NAT1* marker were transformed into the respective TF deletion mutant in all clinical isolate backgrounds, with approximately 2 μg of the TF-containing cassette, 2 μg of the *NAT1* marker cassette, 1 μg of Cas9, and 1 μg of r1 sgRNA DNA cassette. The r1 sgRNA DNA cassette was generated using split-joint PCR with primers “sgRNA/F r1” and “SNR52/R r1”. Heterozygosity or homozygosity at the edited TF locus was determined using the presence or absence of an *r1* scar [35,41] using PCR genotyping with the corresponding “TF Check Up/F” and “r1 check int/R” primers.

### *BRG1* ectopic expression strain construction

To construct *BRG1* ectopic expression strains, we replaced the *MDR1* ORF with varying *BRG1* alleles using our concatemer assembly method [41].

A cassette containing 1642 bp of *BRG1* upstream sequence, the *BRG1* ORF and 712 bp of *BRG1* downstream sequence was amplified from SC5314 genomic DNA using primers “BRG1 1641 5’F->MDR1 up/F” and “BRG1 712 3’R->pNAT 5’/R”, containing concatenating homology to a *NAT1* marker. A *NAT1* marker was then amplified from pNAT using “pNAT for adap/F” and “pNAT 3’R->MDR1 down/R”. The same process was performed with P57055 genomic DNA.

The *BRG1*^SC5314^ or *BRG1*^P57055^ containing cassettes and *NAT1* marker cassette were transformed alongside Cas9 and MDR1 sgRNA DNA cassettes into the P57055 *bcr1*Δ/Δ mutant and P57055 *brg1*Δ/Δ mutant strains. Approximately 2 μg of the *BRG1* containing cassette, 2 μg of the *NAT1* marker cassette, 1 μg of Cas9, and 1 μg of MDR1 sgRNA DNA cassette were included in each transformation mix. The MDR1 sgRNA DNA cassette was generated using split-joint PCR with primers “sgRNA/F MDR1-5” and “SNR52/R MDR1-5”. Integration of either *BRG1* allele at the *MDR1* locus was determined using PCR genotyping with the primers “MDR1 check up/F” and “BRG1 check int/R”. Heterozygosity or homozygosity of *BRG1* integration was determined using PCR genotyping with the primers with the primers “MDR1 check up/F” and “MDR1 check int/R”.

### Biofilm growth and imaging

To assay biofilm formation, strains were inoculated to an OD_600_ of 0.5 from overnight cultures into 2 ml of RPMI + 10% serum containing a 1.5 cm x 1.5 cm silicone square (Bentec Medical Inc., Woodland) in the wells of an untreated 12 well plate. The cells were then incubated in an incubator shaker at 37°C for 90 minutes with mild shaking (60 rpm) to allow for adherence to the silicone square, and following initial adhesion, were washed of non-adherent cells by brief immersion in 2 ml PBS then reintroduced into a new well containing fresh 2 ml of RPMI + 10% serum. Biofilms were then allowed to grow for 24 hours in an incubator shaker at 37°C with mild shaking (60 rpm), before being washed of media and fixed for one hour using a solution of 4% formaldehyde and 2.5% glutaraldehyde in PBS.

Silicone squares from biofilm assays that were not fixed for confocal imaging were soaked in distilled water and agitated to remove the bulk of any adherent biofilm material. Several passes of scrubbing then rinsing in distilled water were then used to remove any remaining adherent material. Silicone squares were then subsequently dried and autoclaved for re-use. To ensure reproducibility, recycled squares were used in all assays in the P57055 background.

Fixed biofilms were stained overnight with Concanavalin A, Alexa Fluor 594 conjugate (Life Technologies) diluted to 25 μg/ml in PBS. Biofilms were then washed once more in PBS to remove any excess dye, then transferred to glass scintillation vials and index matched through subsequent passages through 100% methanol, 50:50 methanol and methyl salicylate solution, and 100% methyl salicylate. Biofilms were then imaged using a slit-scan confocal optical unit on a Zeiss Axiovert 200 microscope with a Zeiss 40x/0.85 NA oil immersion objective. The index matching and imaging are described in greater detail by Lagree et al. [42].

### Hyphal induction assays and imaging

To assay hyphal formation, strains were inoculated to an OD_600_ of 0.5 from overnight cultures into 5 ml of RPMI + 10% serum in glass test tubes. Cells were then grown for 4 hours at 37°C in a roller drum for vigorous agitation. Cells were then collected by centrifugation and fixed with 4% formaldehyde for 15 minutes. Fixed cells were then washed twice in PBS and stained with Calcofluor-white. Stained cells were then imaged using a slit-scan confocal optical unit on a Zeiss Axiovert 200 microscope with a Zeiss C-Apochromat 40x/1.2 NA water immersion objective. Results were then quantified using two metrics: length of hyphal units and ratio of hyphal units to yeast cells. To quantify the length of hyphal units, the distances between septa on hyphae were measured using ImageJ. At least 50 inter-septal distance measurements were taken from 3 separate 112 μm x 83.5 μm fields of view. Hyphal units and yeast cells were then counted using the same fields of view to obtain the ratio of hyphal units to yeast cells.

### RNA extraction and nanostring

For all RNA extractions, strains were inoculated from overnight cultures into 25 ml of RPMI + 10% serum to an OD_600_ of 0.2. Cells were then grown for 4 hours with vigorous shaking (225 rpm) in an incubator shaker then harvested by vacuum filtration and quickly frozen at -80°C until RNA extraction. Three cultures of each strain were grown to provide three biological replicates for Nanostring and RNA-Seq experiments.

RNA extraction and NanoString analysis was performed according to previously published methods [43]. Cell disruption was achieved mechanically using Zirconia beads (Ambion, Fisher Scientific, Waltham), and extraction was performed using a 25:24:1 phenol:chloroform:isoamyl alcohol method combined with a Qiagen RNeasy Mini Kit (Qiagen, Venlo, Netherlands). 25 ng of extracted RNA was added to a nanoString codeset mix and incubated at 65°C for 18 hours, before further binding and washing on a nanoString nCounter Prep Station and scanning on an nCounter digital analyzer. Raw counts were normalized against average total counts with background subtraction. Statistical significance in differential expression was assessed using the Benjamini-Hochberg procedure at a FDR of 0.1.

### RNA-Seq

RNA-Seq was performed on the same RNA samples prepared for Nanostring. Five micrograms of total RNA was incubated with 2 units of TurboDNAse (Invitrogen) in a 50 ul reaction for 15 minutes at 37 degrees C. The RNA was purified by acid phenol-chloroform extraction, and the supernatant containing the RNA was purified over a column and eluted into 15 ul of nuclease free water. Two micrograms of total RNA was used as input for the Lexogen mRNA sense kit v2. The kit was used according to the manufacturer’s instructions for shorter amplicons. Eleven cycles of PCR were performed, incorporating unique barcode indices on each library. The resulting thirty libraries were pooled evenly and subjected to one lane of Illumina sequencing (Novogene), resulting in an average of 16 million reads per library.

Raw fastq reads were trimmed using cutadapt (v 1.9.1) (DOI: https://doi.org/10.14806/ej.17.1.200), with options “-m 42 -a AGATCGGAAGAGC” to remove Illumina 3’ adapter sequence and “-u 10 -u -6” to remove the Lexogen random priming sequences, according to the Lexogen’s instructions. Trimmed reads were mapped using tophat (v 2.0.8) [44] with options “–no-novel-juncs” and “-G” to align to the *C*. *albicans* SC5314 reference genome assembly 22 annotation gff file. Primary alignments were selected using samtools (v 0.1.18) [45] with options “view -h -F 256”. Gene counts were created using “coverageBed” from bedtools (v 2.17.0) [46] with option “-S” to count stranded alignments (as Lexogen reads are reverse complement). The SC5314 release 22 is a phased diploid assembly. RNA-Seq reads mapped to the two alleles of each gene were combined for further analysis. Differential expression was assessed using DEseq2 (v 1.22.1) [47] in R (v 3.5.1) using default options (alpha = 0.05).

### Software

Images were compiled and any adjustments were performed in ImageJ [48]. Single guide RNA sequences were checked for specificity using Cas-OFFinder software [49]. Network graphs were constructed using Cytoscape software [50]. Analyses were performed with Graphpad Prism version 8.00 (Graphpad Software, Inc., La Jolla). Venn diagrams were constructed using Venn Diagrams software (http://bioinformatics.psb.ugent.be/webtools/Venn/).

## Supporting information

S1 Fig Depth of biofilms formed by *C. albicans* clinical isolates. Biofilm depth was quantified from the indicated clinical isolate strains on silicone squares in RPMI + 10% serum at 37°C for 24 hours. Three biological replicates were analyzed for each clinical isolate. Measurements were taken from several positions on each biofilm by confocal microscopy. Values shown are mean depth (SD). Comparisons between isolate biofilm depths were significant (Tukey-Kramer test, P<0.05) except those indicated by a horizontal bar.
(PDF)

S2 Fig Quantification of clinical isolate filamentation. Filamentation capacities of clinical isolate wild-type strains were quantified following hyphal induction. Three technical replicates were performed for each strain. A. Boxplots of the distribution of hyphal unit lengths measured from the indicated clinical isolate background. Whiskers are 1.5IQR. Significant differences in mean hyphal unit length between isolates are indicated (Tukey-Kramer test, ***, P<0.001; ****, P<0.0001). B. Ratio of observed hyphal units to yeast cells in the indicated clinical isolate background. Values are mean (SD). Significant differences in mean hyphal unit: yeast cell ratios are indicated (Tukey-Kramer test, *, P<0.05; **, P<0.01; ****, P<0.0001).
(PDF)

S3 Fig Quantification of clinical isolate TF mutant filamentation. Filamentation capacities of clinical isolate TF mutant strains were quantified following hyphal induction. Three technical replicates were performed for each strain. Top Panel: Boxplots of the distribution of hyphal unit lengths measured from the indicated mutant and clinical isolate background. Whiskers are 1.5IQR. Significance of the difference in mean hyphal unit length between mutant and wild type of the same clinical isolate background is indicated above each value (Dunnett test; *, P<0.05; **, P<0.01; n.s., not significant). Strains in which hyphae were not detected are marked n/a. Bottom Panel: Ratio of observed hyphal units to yeast cells in the indicated mutant and clinical isolate background. Values are mean (SD). Significance of the differences in mean hyphal unit: yeast cell ratios between mutant and wild type of the same clinical isolate background is indicated above each value (Dunnett test; *, P<0.05; **, P<0.01; n.s., not significant). Strains in which hyphae were not detected are marked n/a.
(PDF)

S4 Fig Hyphal formation by reconstituted strains. To validate TF mutant strain filamentation phenotypes, *BCR1*, *UME6*, *BRG1*, *and EFG1* alleles from SC5314 were reconstituted in the corresponding transcription factor mutants in all clinical isolates using our concatemer assembly method [41]. The resultant validation strains were grown in RPMI + 10% serum at 37°C for 4 hours with shaking alongside wild-type and *efg1*Δ/Δ mutant strains in the corresponding clinical isolate backgrounds. Fixed cells were stained with Calcofluor-white and imaged using confocal microscopy. For *efg1*Δ/Δ and *brg1*Δ/Δ mutant strains, filamentation in homozygous validation strains is shown. For *ume6*Δ/Δ mutant validation strains, filamentation in heterozygous validation strains are depicted. For *bcr1*Δ/Δ mutant strains, filamentation in heterozygous validation strains are depicted, except for P75010 in which only homozygous transformants were recovered. Images for *bcr1*Δ/Δ, *brg1*Δ/Δ, and *ume6*Δ/Δ mutant strains are taken from Fig 3 for visual reference. White scale bars in each panel are 20 μm in length.
(PDF)

S5 Fig Quantification of filamentation in P75010 expressing EFG1 or BRG1 alleles from SC5314. P75010 wild-type and P75010 background strains expressing *EFG1* or *BRG1* alleles from SC5314 were quantified following hyphal induction. Three technical replicates were performed for each strain. Left Panel: Boxplots of the distribution of hyphal unit lengths. Whiskers are 1.5IQR. Significance of the difference in mean hyphal unit length is indicated for each background (Dunnett test; ns, not significant; *, P < 0.05). Bottom Panel: Ratio of observed hyphal units to yeast cells. Values are mean (SD). Significance of the differences in mean hyphal unit: yeast cell ratios are indicated for each background (Bonferroni test; **, P < 0.01; ****, P < 0.0001).
(PDF)

S6 Fig Heatmap of glycolytic process gene expression. Heatmap depicts log2 fold change in expression of genes with “glycolytic process” GO annotation. Sample and gene orders reflect hierarchical clustering of gene expression data, with average linkage clustering based on Manhattan distance. Upper (Yellow) and lower bounds (Blue) correspond to a log2 fold change value of 2 and -2 respectively.
(PDF)

S7 Fig Hyphal formation of *BRG1* ectopic expression strains. Wild-type and *BRG1* ectopic expression strains in the P57055 background were assayed for filamentation under planktonic growth conditions. Strains were grown in RPMI + 10% serum at 37°C for 4 hours with shaking. Fixed cells were stained with Calcofluor-white for confocal microscopy. White scale bars in each panel are 20μm in length. **A.** Filamentation in wild-type, *brg1*Δ/Δ, *brg1*Δ/Δ *mdr1*Δ::*BRG1*^*SC5314*^*/mdr1*Δ::*BRG1*^*SC5314*^, and *brg1*Δ/Δ *mdr1*Δ::*BRG1*^*P57055*^*/mdr1*Δ::*BRG1*^*P57055*^ strains. **B.** Filamentation in *bcr1*Δ/Δ and *bcr1*Δ/Δ strains expressing *BRG1*^*SC5314*^ or *BRG1*^*P57055*^. Strains carrying one (heterozygous expression) or two (homozygous expression) copies of *BRG1* alleles from either background were assayed. Two independent isolates are depicted for each case.
(PDF)

S1 Table Nanostring RNA measurements in TF deletion mutants. Three biological replicates were analyzed using Nanostring for each strain. S1A Table contains normalized Nanostring probe counts of each assayed gene from each replicate of each assayed strain. S1B Table contains mean probe counts from the assayed genes in each strain background. S1C Table contains fold changes in gene expression for assayed genes. Fold changes were calculated between TF mutants and wild types of matching clinical isolate backgrounds. S1D Table shows p-values from t-tests between mutant and wild type of matching clinical isolate backgrounds. Values highlighted in yellow are significant according to the Benjamini Hochberg method (FDR = 0.1). S1E Table lists the intersection of direct targets found by Nobile et al. [16], Lassak et al. [23], and assayed genes. S1F Table contains raw Nanostring probe count data. S1G Table contains lists of TF-responsive genes in each clinical isolate background.
(XLSX)

S2 Table Numbers of genes with significantly altered expression in each TF mutant. The number of affected genes per mutant in each clinical isolate background are shown, based only on significant difference between mutant and clinical isolate wild type in mean gene expression.
(XLSX)

S3 Table RNA-Seq RNA measurements. S3A Table contains fold change in expression values and padj values from DeSeq2 analysis of un-normalized counts comparing *efg1*Δ/Δ mutant and matched clinical isolate wild-type strains. Three biological replicates were analyzed using RNA-Seq for each *efg1*Δ/Δ mutant and clinical isolate. S3B Table contains RPKM values from each replicate of the assayed strains. S3C Table lists the Efg1-responsive genes in each clinical isolate background divided between Efg1-repressed (up in mutant) and Efg1-activated (down in mutant) targets. Genes with at least a significant (padj <0.05), 2 fold difference in expression were called as targets in any given background. S3D Table lists the genes unique to each of the indicated intersecting sets of backgrounds, as well as genes uniquely differentially expressed in each background.
(XLSX)

S4 Table GO term descriptors for Efg1-responsive genes. GO Term Finder results are shown for the listed sets of Efg1-responsive genes. Results are separated by process, function, and component.
(XLSX)

S5 Table Quantification of hyphal formation in *BRG1* ectopic expression strains. Statistics regarding ratio of hyphal unit to yeast cells and hyphal unit length for P57055 wild-type and *BRG1* ectopic expression strains are listed. Three technical replicates were analyzed. Significance of comparisons against wild type and *bcr1*Δ/Δ or *brg1*Δ/Δ mutants are shown, (Tukey-Kramer test; **, P < 0.01; ****, P < 0.0001).
(XLSX)

S6 Table GO term descriptors for gene expression differences between wild-type strains. S6A Table contains lists of genes whose expression in clinical isolate strains relative to SC5314 correlated or anti-correlated with BRG1 expression relative to SC5314. S6B Table contains GO Term Finder results for the listed sets of genes found in S6A Table. S6C Table contains lists of genes differentially expressed in clinical isolate strains (2-fold difference in expression, padj < 0.05). S6D Table contains GO Term Finder results for the listed sets of genes found in S6C Table.
(XLSX)

S7 Table Strains. List of all *C*. *albicans* strains used in this study.
(XLSX)

S8 Table Primers and Plasmids. S8A Table contains a list of all primers used in this study. S8B Table contains a list of all plasmids used in this study.
(XLSX)
