# Correction: Circuit diversification in a biofilm regulatory network

**DOI:** 10.1371/journal.ppat.1007966

**Published:** 2019-07-19

**Authors:** The PLOS Pathogens Staff

## Notice of Republication

Supplementary Tables S5-S8 were mislabelled due to errors introduced during the typesetting process. The publisher apologizes for the errors. This article was republished on July 5, 2019 to correct for these errors. Please download this article again to view the correct version.
