# Supplementary figures and images for: Circuit diversification in a biofilm regulatory network
Source: PLoS Pathog. 2019 May 22;15(5):e1007787. doi: 10.1371/journal.ppat.1007787 (PMC6530872; doi:10.1371/journal.ppat.1007787)

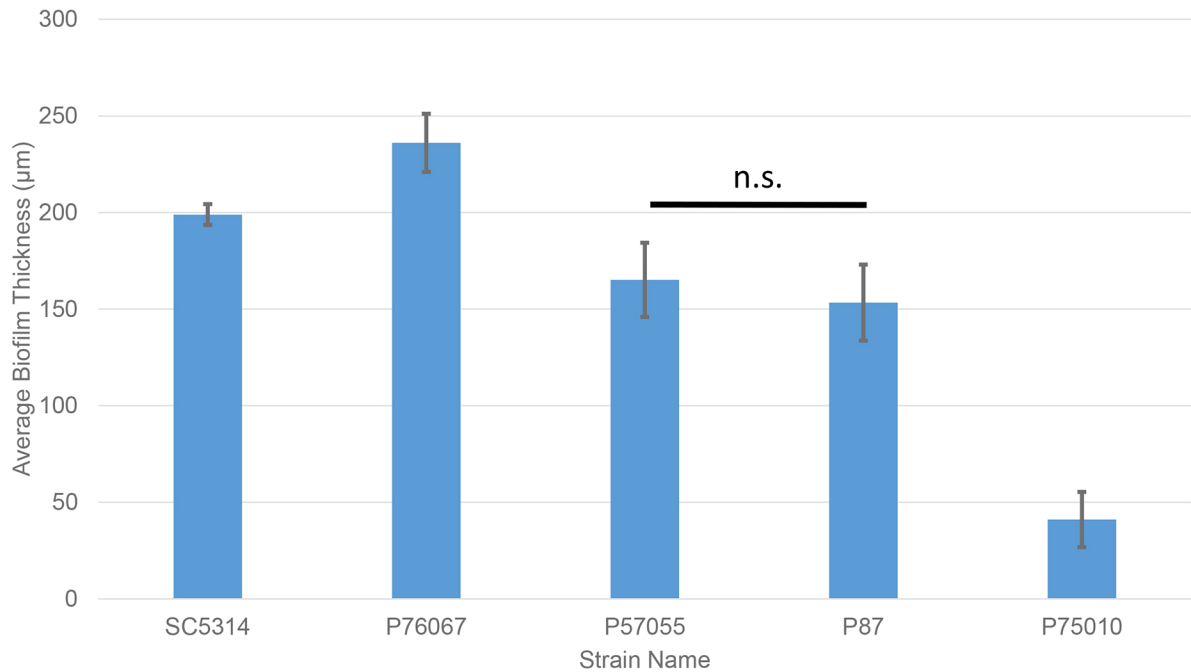

Supplement: S1 Fig — Biofilm depth was quantified from the indicated clinical isolate strains on silicone squares in RPMI + 10% serum at 37°C for 24 hours. Three biological replicates were analyzed for each clinical isolate. Measurements were taken from several positions on each biofilm by confocal microscopy. Values shown are mean depth (SD). Comparisons between isolate biofilm depths were significant (Tukey-Kramer test, P<0.05) except those indicated by a horizontal bar. (PDF) [file ppat.1007787.s001.pdf]

A

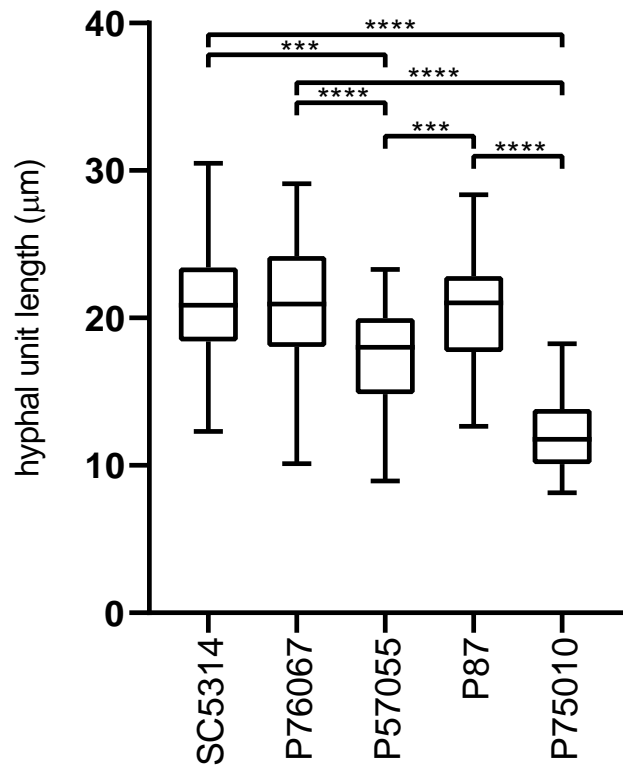

B

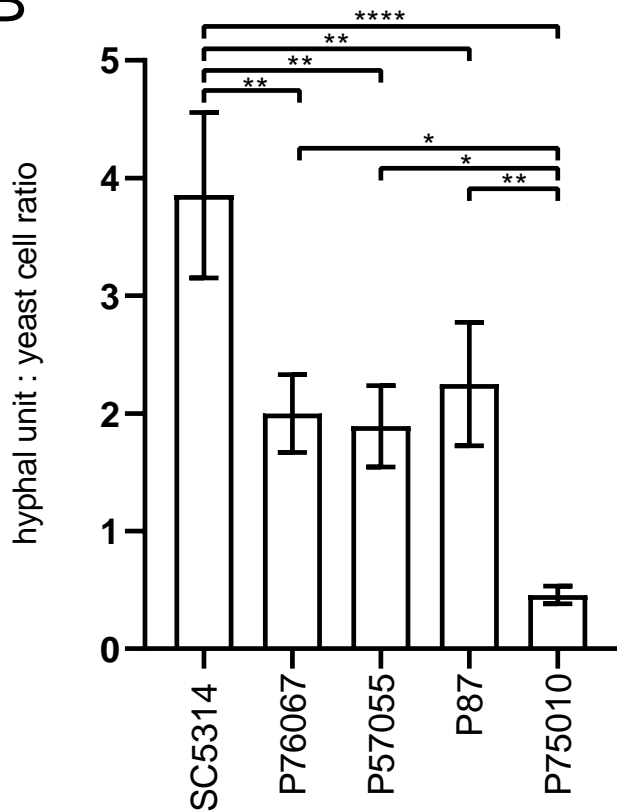

Supplement: S2 Fig — Filamentation capacities of clinical isolate wild-type strains were quantified following hyphal induction. Three technical replicates were performed for each strain. A. Boxplots of the distribution of hyphal unit lengths measured from the indicated clinical isolate background. Whiskers are 1.5IQR. Significant differences in mean hyphal unit length between isolates are indicated (Tukey-Kramer test, ***, P<0.001; ****, P<0.0001). B. Ratio of observed hyphal units to yeast cells in the indicated clinical isolate background. Values are mean (SD). Significant differences in mean hyphal unit: yeast cell ratios are indicated (Tukey-Kramer test, *, P<0.05; **, P<0.01; ****, P<0.0001). (PDF) [file ppat.1007787.s002.pdf]

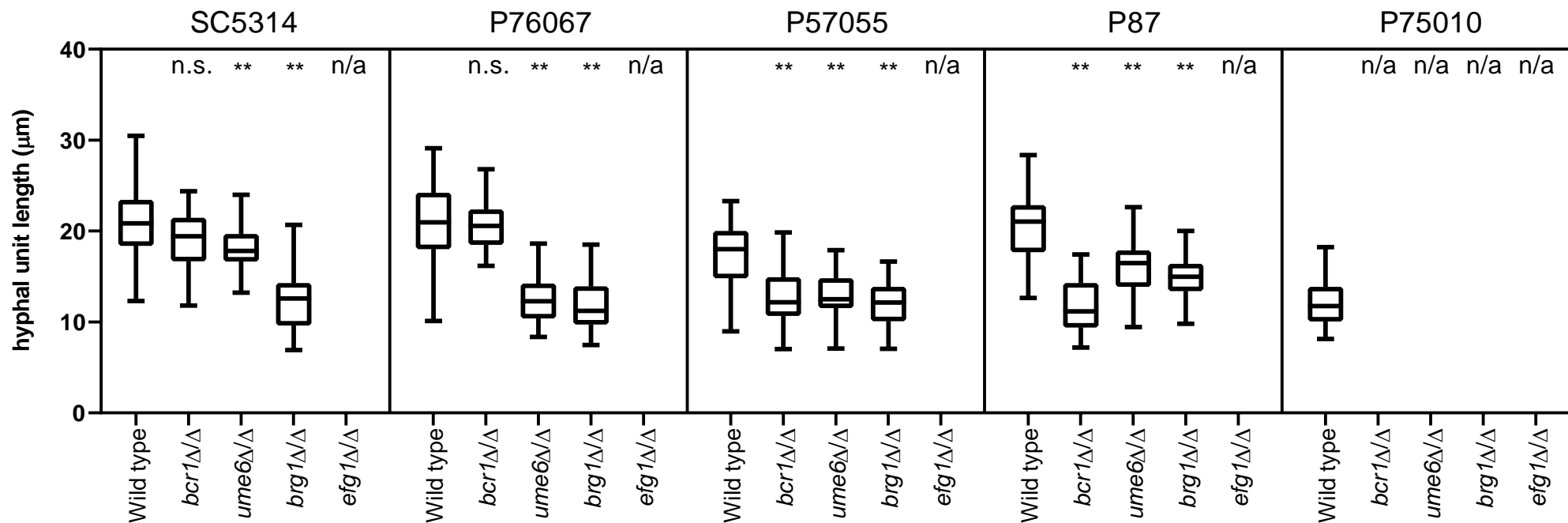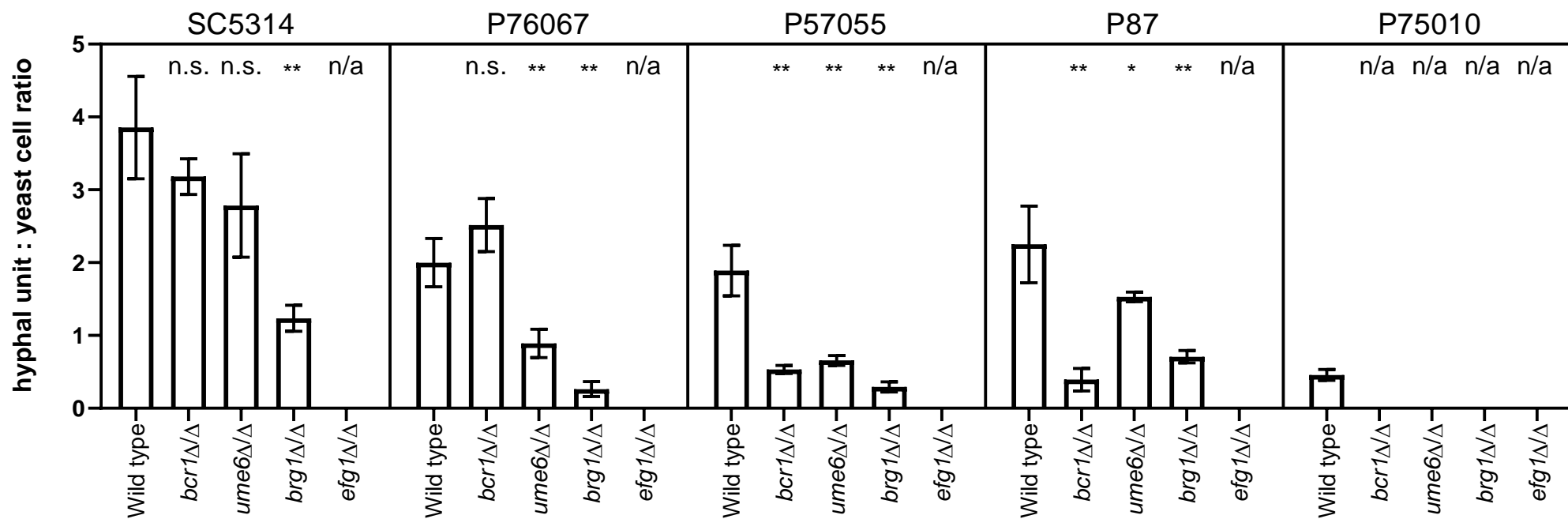

Supplement: S3 Fig — Filamentation capacities of clinical isolate TF mutant strains were quantified following hyphal induction. Three technical replicates were performed for each strain. Top Panel: Boxplots of the distribution of hyphal unit lengths measured from the indicated mutant and clinical isolate background. Whiskers are 1.5IQR. Significance of the difference in mean hyphal unit length between mutant and wild type of the same clinical isolate background is indicated above each value (Dunnett test; *, P<0.05; **, P<0.01; n.s., not significant). Strains in which hyphae were not detected are marked n/a. Bottom Panel: Ratio of observed hyphal units to yeast cells in the indicated mutant and clinical isolate background. Values are mean (SD). Significance of the differences in mean hyphal unit: yeast cell ratios between mutant and wild type of the same clinical isolate background is indicated above each value (Dunnett test; *, P<0.05; **, P<0.01; n.s., not significant). Strains in which hyphae were not detected are marked n/a. (PDF) [file ppat.1007787.s003.pdf]

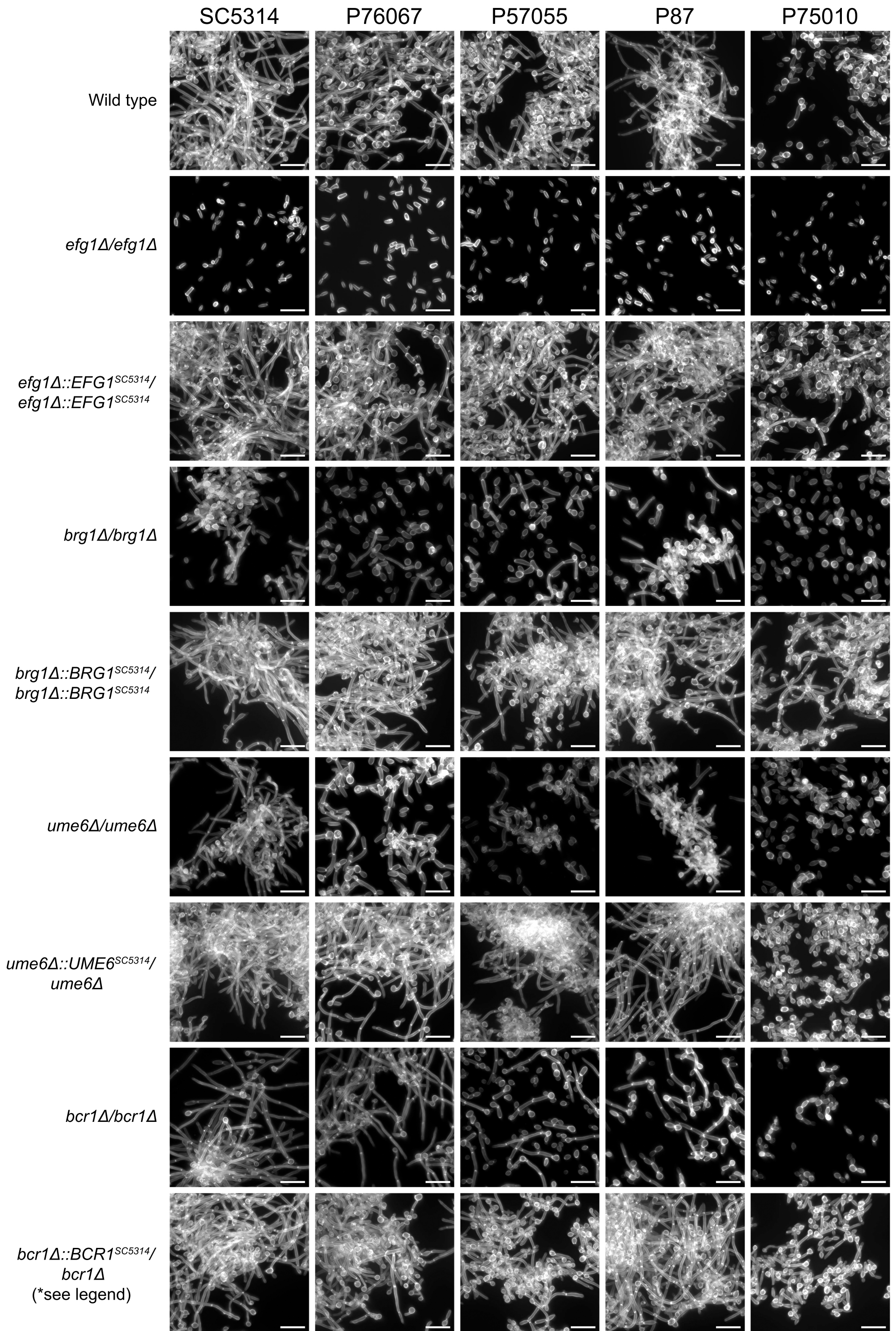

Supplement: S4 Fig — To validate TF mutant strain filamentation phenotypes, BCR1, UME6, BRG1, and EFG1 alleles from SC5314 were reconstituted in the corresponding transcription factor mutants in all clinical isolates using our concatemer assembly method [41]. The resultant validation strains were grown in RPMI + 10% serum at 37°C for 4 hours with shaking alongside wild-type and efg1Δ/Δ mutant strains in the corresponding clinical isolate backgrounds. Fixed cells were stained with Calcofluor-white and imaged using confocal microscopy. For efg1Δ/Δ and brg1Δ/Δ mutant strains, filamentation in homozygous validation strains is shown. For ume6Δ/Δ mutant validation strains, filamentation in heterozygous validation strains are depicted. For bcr1Δ/Δ mutant strains, filamentation in heterozygous validation strains are depicted, except for P75010 in which only homozygous transformants were recovered. Images for bcr1Δ/Δ, brg1Δ/Δ, and ume6Δ/Δ mutant strains are taken from Fig 3 for visual reference. White scale bars in each panel are 20 μm in length. (PDF) [file ppat.1007787.s004.pdf]

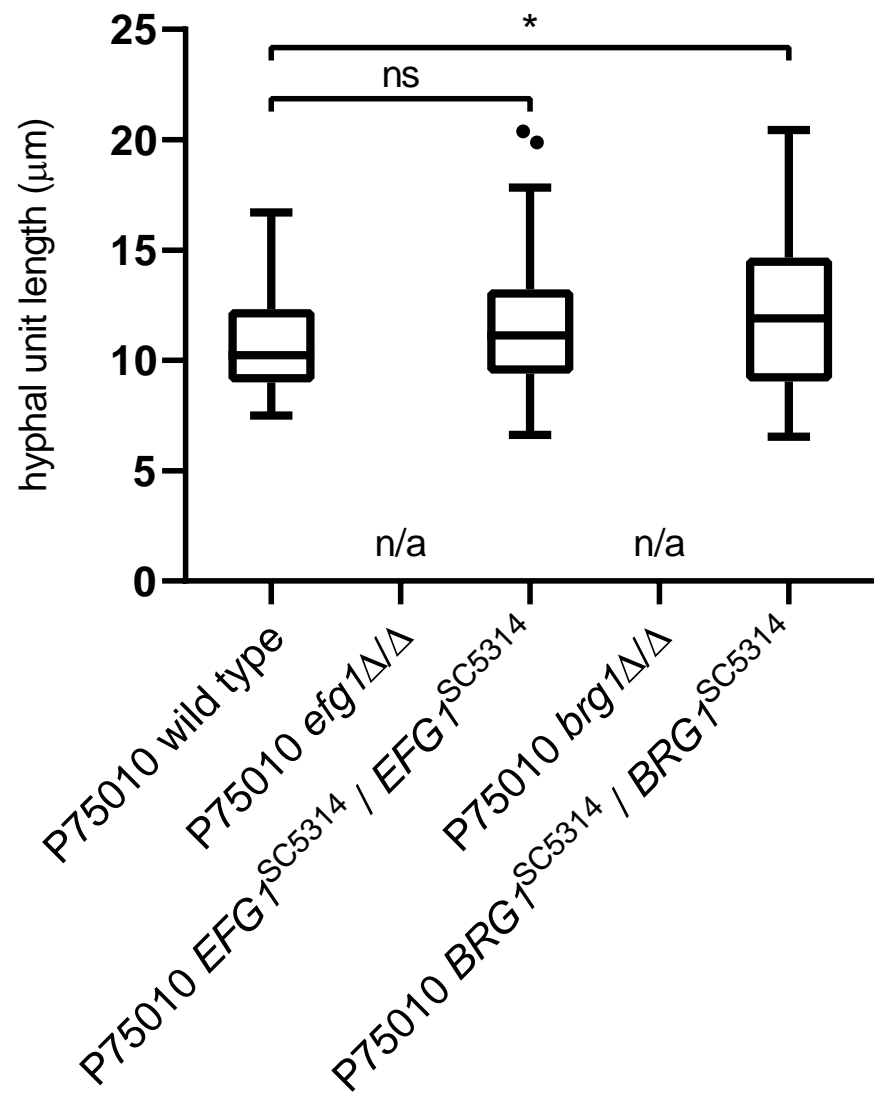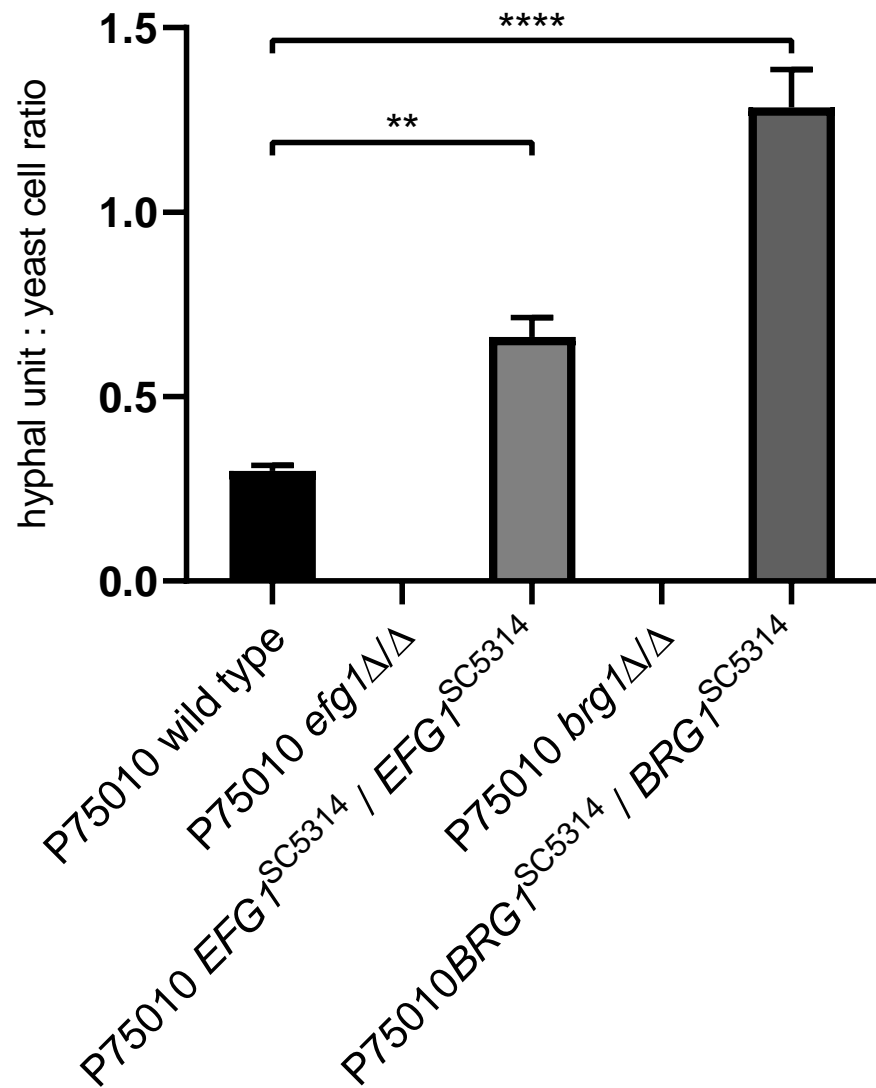

Supplement: S5 Fig — P75010 wild-type and P75010 background strains expressing EFG1 or BRG1 alleles from SC5314 were quantified following hyphal induction. Three technical replicates were performed for each strain. Left Panel: Boxplots of the distribution of hyphal unit lengths. Whiskers are 1.5IQR. Significance of the difference in mean hyphal unit length is indicated for each background (Dunnett test; ns, not significant; *, P < 0.05). Bottom Panel: Ratio of observed hyphal units to yeast cells. Values are mean (SD). Significance of the differences in mean hyphal unit: yeast cell ratios are indicated for each background (Bonferroni test; **, P < 0.01; ****, P < 0.0001). (PDF) [file ppat.1007787.s005.pdf]

-2.0

0.0

2.0

P76067 *efg1Δ* / WTP75010 *efg1Δ* / WTP87 *efg1Δ* / WTP57055 *efg1Δ* / WTSC5314 *efg1Δ* / WT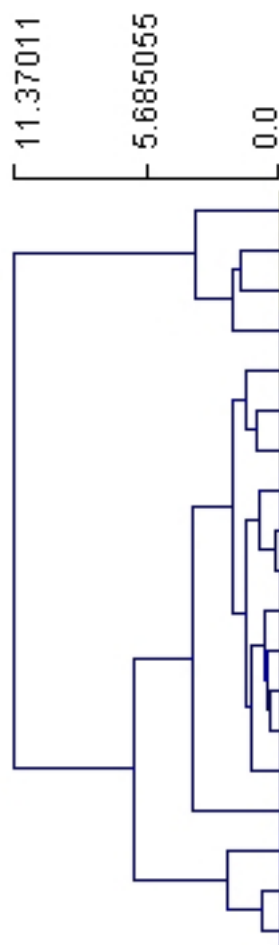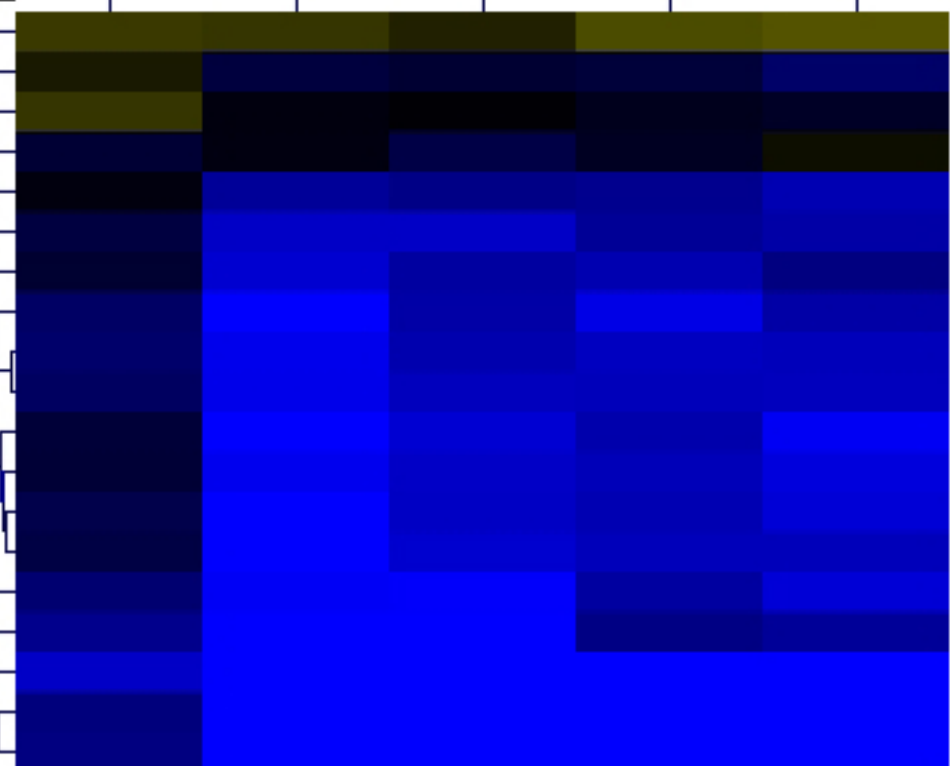

Supplement: S6 Fig — Heatmap depicts log2 fold change in expression of genes with “glycolytic process” GO annotation. Sample and gene orders reflect hierarchical clustering of gene expression data, with average linkage clustering based on Manhattan distance. Upper (Yellow) and lower bounds (Blue) correspond to a log2 fold change value of 2 and -2 respectively. (PDF) [file ppat.1007787.s006.pdf]

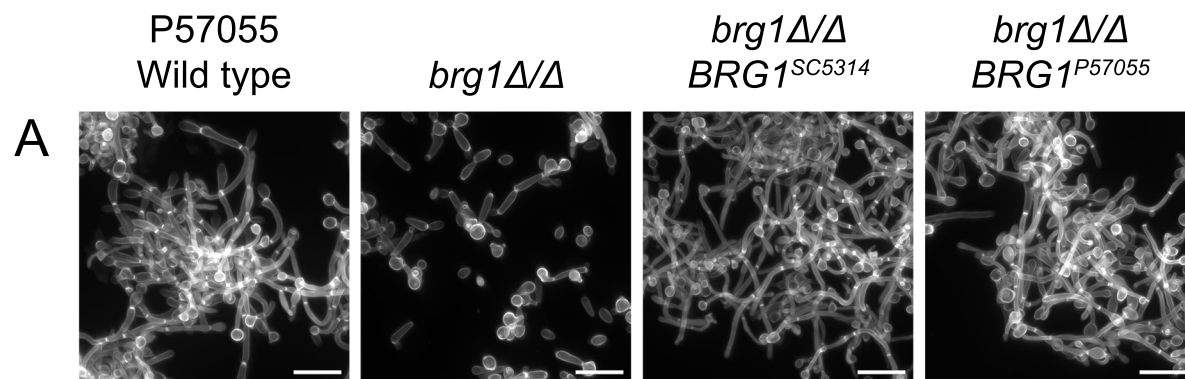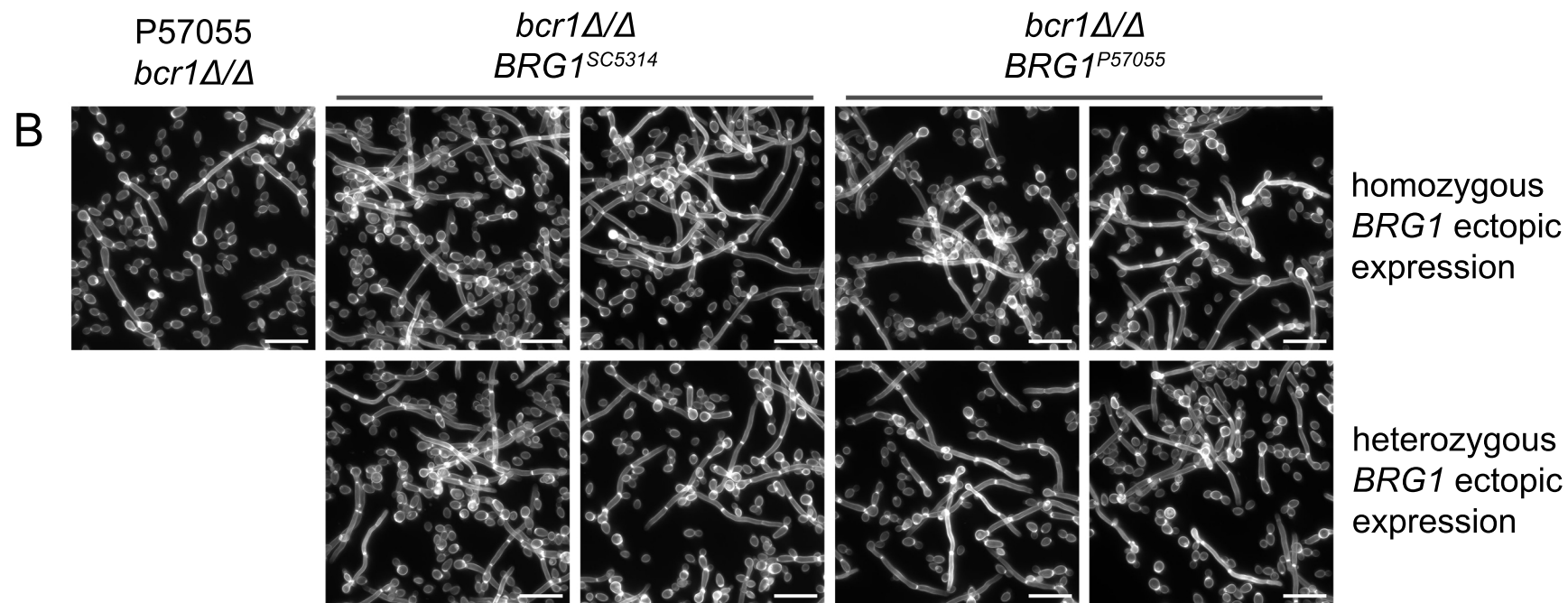

Supplement: S7 Fig — Wild-type and BRG1 ectopic expression strains in the P57055 background were assayed for filamentation under planktonic growth conditions. Strains were grown in RPMI + 10% serum at 37°C for 4 hours with shaking. Fixed cells were stained with Calcofluor-white for confocal microscopy. White scale bars in each panel are 20μm in length. A. Filamentation in wild-type, brg1Δ/Δ, brg1Δ/Δ mdr1Δ::BRG1SC5314/mdr1Δ::BRG1SC5314, and brg1Δ/Δ mdr1Δ::BRG1P57055/mdr1Δ::BRG1P57055 strains. B. Filamentation in bcr1Δ/Δ and bcr1Δ/Δ strains expressing BRG1SC5314 or BRG1P57055. Strains carrying one (heterozygous expression) or two (homozygous expression) copies of BRG1 alleles from either background were assayed. Two independent isolates are depicted for each case. (PDF) [file ppat.1007787.s007.pdf]
